# Zfp697/ZNF697 is an RNA-binding protein that modulates TGF-β1 signaling and hepatic inflammation in liver disease

**DOI:** 10.1016/j.molmet.2026.102457

**Published:** 2026-10-14

**Authors:** Stine M. Præstholm, Jorge C. Correia, Rafael Hoyos Justiniano, Alisson L. da Rocha, Aurel B. Leuchtmann, Tadas Sereiva, Margareta Porsmyr-Palmertz, Kathrine Rothamel, Dorota Kaminska, Ranjani Sri Ganji, David Potěšil, Zbyněk Zdráhal, Jussi Pihlajamäki, Gene W. Yeo, Jorge L. Ruas

**Affiliations:** 1 Molecular and Cellular Exercise Physiology, Department of Physiology and Pharmacology, Biomedicum, Karolinska Institutet, Stockholm, SE-171 77, Sweden; 2 Department of Pharmacology and Stanley & Judith Frankel Institute for Heart & Brain Health, University of Michigan Medical School, Ann Arbor, MI, 48109, USA; 3 Neuroscience Graduate Program, University of Michigan, Ann Arbor, MI, USA; 4 Cell and Molecular Biology Program, University of Michigan, Ann Arbor, MI, USA; 5 Department of Cellular and Molecular Medicine, University of California, San Diego, La Jolla, CA, USA; 6 Institute of Public Health and Clinical Nutrition, University of Eastern Finland, Kuopio Campus, 70210 Kuopio, Finland; 7 Department of Medicine, Endocrinology and Clinical Nutrition, Kuopio University Hospital, Wellbeing Services County of North Savo, 70210 Kuopio, Finland; 8 Central European Institute of Technology, CEITEC, Masaryk University, Kamenice 5, 625 00 Brno, Czech Republic; 9 National Centre for Biomolecular Research, Faculty of Science, Masaryk University, Kamenice 5, 625 00 Brno, Czech Republic; 10 Biomedical Sciences Graduate Program, University of California, San Diego, La Jolla, CA, USA; 11 Institute for Genomic Medicine, University of California, San Diego, La Jolla, CA, USA

**Keywords:** Liver disease, Inflammation, MASLD, TGF-β1, Zinc finger protein, RNA-Binding

## Abstract

Liver diseases driven by metabolic dysfunction, inflammation, and fibrosis such as metabolic dysfunction-associated steatotic liver disease (MASLD), including metabolic dysfunction-associated steatohepatitis (MASH), and acute liver injury represent a growing global health burden. The zinc finger protein Zfp697 (ZNF697 in humans) is a newly identified regulator of inflammation and regeneration in skeletal muscle. Here, we characterize the role of liver Zfp697 and show that its expression is increased in multiple animal models of liver disease. Indeed, *Zfp697/ZNF697* transcript levels show a strong positive correlation with several markers of liver inflammation and fibrosis. In line with that, in human and mouse hepatocytes TGF-β1 increases *Zfp697* expression, which in turn regulates genes involved in TGF-β1 signaling, chemokine secretion, and immune cell recruitment. In mice, hepatocyte-specific *Zfp697* deletion improves glucose tolerance and is associated with the absence of MASH-induced lean mass loss but does not confer uniform hepatoprotection. Biochemical purification of Zfp697 protein interactors and enhanced crosslinking and immunoprecipitation (eCLIP) analyses reveal that Zfp697 interacts with other RNA-binding proteins and associates with over 1,000 RNAs in mouse liver. Zfp697-associated RNAs include several transcript types, with a large representation of mRNAs which group in pathways related to transcriptional regulation**,** PI3K/AKT signaling, and cell differentiation. These findings identify Zfp697 as a novel RNA-interacting protein and modulator of TGF-β signaling in the liver and provide a basis for further investigation of its role in liver disease.

## Highlights

• Zinc finger protein Zfp697/ZNF697 is associated with liver injury and disease.
• Zfp697 regulates genes related to inflammation and ECM remodeling.
• Zfp697 modulates the hepatic transcriptional response to TGF-β1 signaling.
• Hepatocyte-specific Zfp697 knockout alleviates MASH phenotype in mice.
• Zfp697 associates with RNA-binding proteins and liver-specific RNAs.

## Graphical abstract

**Figure:**
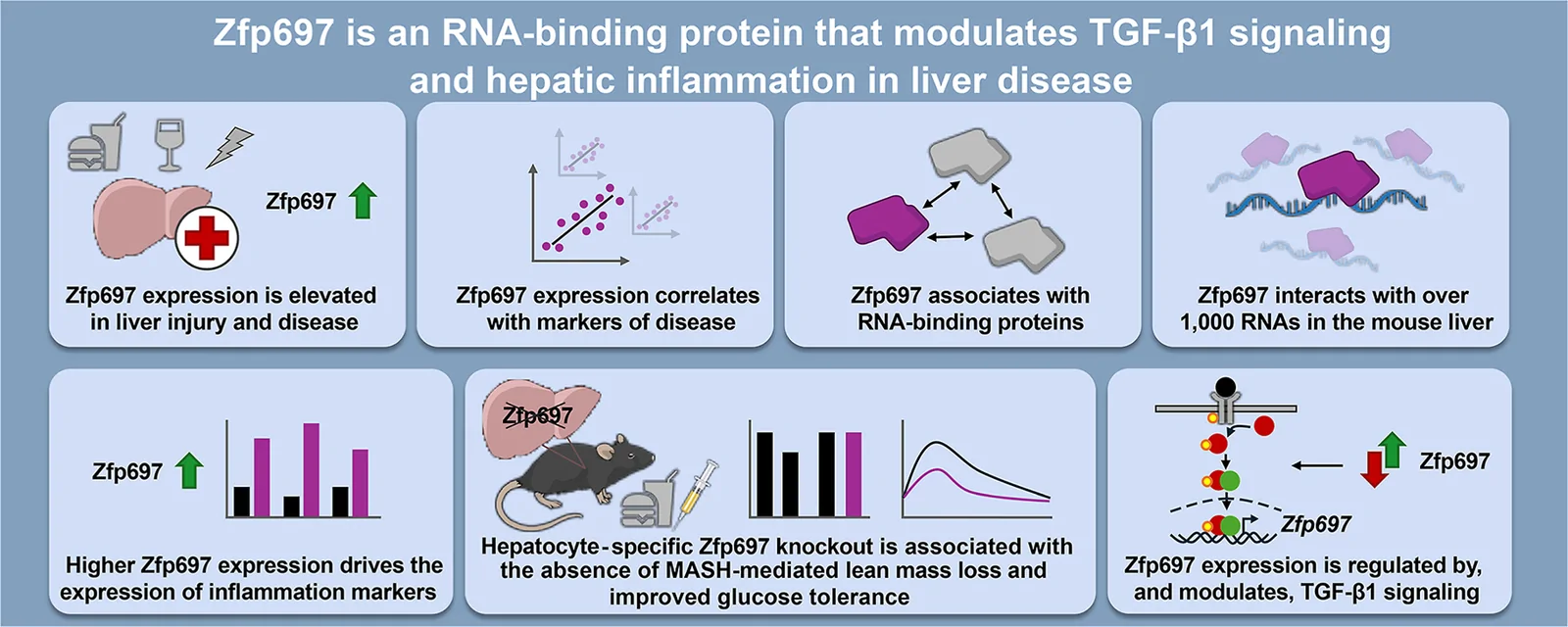

## 1 Introduction

Liver diseases and injuries are characterized by several key pathological processes such as inflammation, oxidative stress, metabolic dysregulation, cell death, tissue remodeling, fibrosis, and regeneration, each regulated by complex mechanisms [1], [2], [3]. These liver conditions pose a significant global health burden, with metabolic dysfunction-associated steatotic liver disease (MASLD) including metabolic dysfunction-associated steatohepatitis (MASH) in particular rising in prevalence in parallel with increasing rates of obesity, metabolic syndrome, and type 2 diabetes [4]. As these diseases continue to escalate worldwide, there is an urgent need to uncover the underlying molecular mechanisms driving disease onset, progression, and recovery. Understanding these mechanisms could ultimately lead to the identification of novel therapeutic targets to mitigate or reverse liver disease.

Zinc finger proteins (ZFPs) are a large and diverse class of proteins with a wide range of molecular functions and mechanisms of action [5] including RNA binding, which is increasingly recognized as a feature of many members of the family. Some ZFPs are associated with the development and/or resolution of inflammation [6], [7], [8], [9] including Zfp697 (in mice; ZNF697 in humans), a previously under-characterized ZFP, recently identified by our lab as a regulator of skeletal muscle inflammation and regeneration [10]. More recently, ZNF697 was found to be part of a tumor-associated macrophage gene signature in lung adenocarcinoma, where its expression was associated with poorer survival outcomes [11]. As chronic, unresolved inflammation has been identified as a key marker and contributor to the progression of liver diseases, including MASLD and MASH, we sought to investigate the potential role of Zfp697 in liver inflammation and disease.

In this study, we investigated the role of liver Zfp697 and its potential as a therapeutic target for hepatic disease. We show that *Zfp697* and *ZNF697* levels are increased following both acute and chronic liver injury, and across multiple animal liver disease models. *Zfp697/ZNF697* expression in liver cells is responsive to inflammation- and injury-associated signals, including TGF-β1, IFN-γ, IL-6, and oxidative stress. We demonstrate that *Zfp697* and *ZNF697* expression correlates with disease marker genes including key chemokine and inflammatory genes in different mouse models of liver disease and injury, as well as in human liver disease patients. Additionally, we show that *Zfp697* and *ZNF697* are regulated by the canonical TGF-β1 pathway and that their expression modulates TGF-β1-induced SMAD2 phosphorylation and the hepatic transcriptional response to TGF-β1. Interestingly, we find that mice lacking Zfp697 specifically in hepatocytes do not exhibit the MASH-associated loss of lean mass or glucose intolerance observed in WT mice, with RNA-seq data showing alterations in metabolic, inflammatory, and extracellular matrix-associated signaling pathways. We find that Zfp697 interacts with key RNA-binding proteins in the liver and associates with multiple RNAs in a context-specific manner. Our findings establish Zfp697 as a novel modulator of hepatic inflammation and MASH disease response.

## 2 Methods

### 2.1 Data from kuopio obesity surgery (KOBS) study

The Kuopio Obesity Surgery (KOBS) cohort has been described previously [12], [13]. This study included 265 unrelated adults from KOBS who underwent bariatric surgery at Kuopio University Hospital, with liver biopsies collected during surgery. Liver histology (steatosis, lobular inflammation, ballooning, fibrosis) was scored, and participants were classified as Control (healthy liver), metabolic-associated steatotic liver (MASL), or metabolic-associated steatohepatitis (MASH).

Gene-level read counts were obtained from liver RNA-seq aligned with STAR (2-pass) and quantified with featureCounts using GENCODE v29 annotation. Trimmed mean of M-values (TMM) normalization factors were estimated from raw counts in edgeR and applied to effective library sizes before transcripts per million (TPM) calculation. TPM values were log2-transformed, and expression was residualized by regressing out 3′ bias, alignment rate, RNA integrity number (RIN), body mass index (BMI), age, and sex.

Biweight midcorrelation (bicor; WGCNA) was computed between ZNF697 and each candidate gene across samples, with two-sided p-values for each correlation. ZNF697 expression was compared across Control, MASL, and MASH using Kruskal–Wallis (KW), followed by Benjamini-Hochberg (BH)-adjusted pairwise Wilcoxon tests.

### 2.2 Primary human hepatocytes

Primary human hepatocytes were provided by CLINTEC, KI, Unit for Transplantation Surgery, Karolinska University Hospital Huddinge, SE-141 86 Stockholm, Sweden. Cells were cultured in William's E medium (Sigma–Aldrich cat: W1878) supplemented with 2 mM glutamine, 20 mM HEPES, 1 nM insulin (Novo Nordisk Actrapid), 10 μM dexamethasone, 50 μg/mL Gentamicin, and 0.05 μg/mL amphotericin B. Cells were used in experiments and harvested within 48 h after seeding.

### 2.3 Animal experiments

All animal experiments were approved by the regional animal ethics committee of Northern Stockholm, Sweden (ethical permits no. 4039–2018 & 4359–2020). C57BL/6 J and C57BL/6 N mice were purchased from Janvier Labs and acclimated to the animal facility being used in experiments. Mice were housed in groups of 2–5 in ventilated cages at 22–23 °C on a 12 h light/dark cycle with water and standard chow diet (SDS Diets cat: SDS CRM(P)) or (SAFE cat: CRM(P)) *ad libitum*. Only healthy age- and sex-matched littermates were used in experiments. Zfp697 floxed mice were generated on a C57BL/6N background by Biocytogen (USA) inserting loxP sites surrounding exon 3 as described in Correia et al., 2024 [10], and bred in house on a C57BL/6J background. Hepatocyte-specific KO mice were generated by breeding floxed Zfp697 mice with mice expressing Cre recombinase under the control of the albumin promoter [14].

### 2.4 Methionine- and choline-deficient (MCD) diet

Male C57BL/6J mice, 16 weeks of age, were fed an MCD l-amino acid diet (Research Diets cat: A02082002B) or control diet (Research Diets cat: A02082003B), n = 6. Body weights were monitored daily, and the experiment was terminated after 7 days as mice had lost more than 15% of their body weight. Livers were isolated, weighed, and used for RNA extraction.

### 2.5 Carbon tetrachloride injections

Male C57BL/6J mice, 10 weeks of age, received a single intraperitoneal injection of 25 μl 20% CCl_4_ (Sigma–Aldrich cat: 270,652) diluted in corn oil (Sigma–Aldrich cat: C8267) or corn oil as control, n = 4–5. Solutions were heated to 37 °C before injection. Mice were sacrificed 16 h post injection, and livers were isolated for RNA analysis.

### 2.6 Adenoviral injections

C57BL/6J male mice, 9 weeks of age, were administered intravenous injections of 6.68 × 10^9^ IFU/mouse Ad-FLAG-Zfp697 or Ad-GFP into the lateral tail veins. Mice were monitored, and livers were isolated after 7 days.

### 2.7 MASH diet

Male Zfp697 HepKO and floxed control mice, 11 weeks of age, were randomly subjected to a MASH-inducing protocol for 12 weeks as described in Tsuchida et al., 2018 [15]. Specifically, mice were fed a Western diet (Envigo cat: TD.120,528) with high cholesterol (1.25%). % kcal from: 15.2 protein, 42.7 carbohydrate, and 42.0 fat. This was combined with drinking water containing 18.9 g/L glucose and 23.1 g/L fructose, sterile-filtered. In addition, mice were injected intraperiotoneally with CCl_4_ (Sigma–Aldrich cat: 270652) dissolved in corn oil (Sigma–Aldrich cat: C8267) at a concentration of 0.32 μg/g body weight weekly. CCl_4_ solution and corn oil were heated to 37 °C before injection. Control mice were fed a standard chow diet (SDS diets cat: SDS CRM(P)), given regular drinking water, and received weekly injections with corn oil. The weekly injections were performed on Tuesdays or Wednesdays, while during the week of TSE, they were performed right after TSE on Friday.

### 2.8 Body composition by EchoMRI

Total, lean, and fat mass of Zfp697 HepKO mice on MASH-inducing protocol or controls were determined using an EchoMRI-100 instrument. MRI was performed just before indirect calorimetry, and the data were used in the downstream ANCOVA analysis of TSE data as covariates.

### 2.9 Indirect calorimetry

Indirect calorimetry was performed on Zfp697 HepKO mice and controls 10 weeks into the MASH-inducing protocol. Mice were single-caged and placed in metabolic chambers (TSE Systems Phenomaster) for 5 days under the following chamber conditions: temperature 24.2–25.4 °C, light at day: 60.1% and at night: 0%. Temperature of the room was 22.6–25 °C. The dark photoperiod 18:00–06:00. Mice had free access to their diets; either chow (SDS diets cat: SDS CRM(P)) or Western diet (Envigo cat: TD.120528) with the metabolizable energy content of 2.674 kcal/g or 4.5 kcal/g, respectively. Mice had also free access to drinking water, or for the MASH mice, sugar-rich water with the energy content of 0.1633 kcal/mL. TSE Systems Phenomaster collected data every 3 min. Data from the first 31 h were excluded to account for the mice's acclimatization period and initial stress response to the new environment. Data from 32 to 79 h, constituting 2 light-phases and 2-dark phases, were used for data analysis. CalR [16] was used for the initial data analysis, hourly averages, interpretation and statistical analysis. Energy expenditure (EE) was calculated using the Weir equation [17]: EE (kcal/h) = 0.06 *x* [3.941 *x* VCO_2_ (mL/min) + 1.106 *x* VCO_2_ (mL/min)].

ANCOVA analysis was performed using total, lean, and fat mass as covariates.

### 2.10 Glucose tolerance test

At the end of the MASH-inducing protocol, mice were fasted for 5 h starting at 10 AM. Mice were weighed and injected intraperitoneally with 10 μl/g body weight of 20% glucose dissolved in phosphate buffered saline (PBS). Glucose levels were measured from a small tail bleed before injection and 15, 30, 60, and 120 min after glucose administration using the glucometer Abbott Diabetes Care FreeStyle Precision Neo and glucose strips (Abbott cat: 98780–75).

### 2.11 Plasma and tissue collection

At the end of the animal experiments, mice were placed under isoflurane anesthesia and euthanized by decapitation or cervical dislocation. Blood was collected into EDTA-coated tubes and kept on ice. Plasma was obtained by centrifugation at 2000 x *g* for 10 min and stored at −80 °C until analysis at Klinisk kemiska laboratoriet SLU Universitetsdjursjukhuset, SE-756 51 Uppsala, Sweden. Livers were collected and weighed, and a small piece from the left lateral lobe was saved for RNA analysis, snap-frozen in liquid nitrogen and stored at −80 °C. The left medial lobe was fixed in 4% PFA at 4 °C for 24 h, followed by PBS washes and storage in PBS containing 0.04% NaN_3_.

### 2.12 Liver histology and picrosirius red staining

Fixed liver samples were gradually transferred to 30% sucrose and incubated for 24–48 h. Excess sucrose was removed from the tissue before the samples were placed in molds, embedded in O.C.T. (VWT Q-Path Chemicals cat: 00411243), and frozen on dry ice. Samples were stored at −80 °C until cryosectioning. Tissue slides were washed under running distilled H_2_O and incubated in staining solution: 0.1% Sirius red (Direct Red 80) (Sigma–Aldrich cat: 365548) in saturated picrosirius acid (Sigma–Aldrich cat: P6744-1 GA) for 1 h at room temperature. Slides were dipped twice in water containing 0.5% acetic acid (Sigma–Aldrich cat: A6283), dehydrated by three dips in absolute EtOH, and cleared for 2 min in xylene before mounting with Pertex (Histolab cat: 00840–05) and imaging.

### 2.13 Primary mouse hepatocyte isolation

Male C57BL/6N or C57BL/6J mice, 8–12 weeks of age, were used for the primary hepatocyte isolation. Mice were anesthetized with isoflurane, and the liver, inferior vena cava, and portal vein were exposed. Livers were perfused with 37 °C warm HBSS (Gibco cat: 14175–053) containing 21.4 mM NaHCO_3_ and 684.4 μM EDTA at a flow rate of 7 mL/min through vena cava and the portal vein was cut for drainage. When cleared, the perfusion media was changed to liver digest media (Thermo Fisher cat: 17703034), pH 7.4, for 10 min. The digested liver was transferred to a petri dish, and hepatocytes were liberated by gently removing the Glisson's capsule. The cell suspension was passed through a 70 μM cell strainer, followed by washes in cold plating media: DMEM (Gibco cat: 31966) containing 10% fetal bovine serum (FBS), 1% P/S, 1 μM dexamethasone, and 0.1 μM insulin, centrifuging at 50 x *g* at 4 °C. Percoll (Sigma–Aldrich cat: P1644) was used for density gradient centrifugation to further purify the hepatocytes. Cells were inspected and counted, and only preparations with >85% viability were used in experiments. Cells were seeded on collagen-coated cell culture plates (VWR cat: 734–0295) in plating media and allowed to attach before switching to maintenance media: DMEM (Gibco cat: 31966) supplemented with 0.2% bovine serum albumin (BSA), 1% P/S, 0.1 μM dexamethasone, 1 nM insulin). Cells were used for experiments within 48 h from seeding.

### 2.14 Cell cultures

Huh7 cells were cultured in DMEM (Gibco cat: 10,569–010) supplemented with 10% FBS and 1% P/S. LX-2 cells were cultured in DMEM (Sigma–Aldrich cat: D5671) supplemented with 10% FBS, 1% P/S, and 1x Glutamine (Thermo Scientific cat: 25030024).

### 2.15 Cell treatments

Cells were treated for 24 h with 10 ng/mL TGF-β1 (R&D Systems cat: 7666-MB-005), 10 μM Smad3 inhibitor (SIS3) (AdooQ BioScience cat: A16065), 5 μM SB525334 (Tocris cat: 3211) for 24 h, 100, 200, or 400 μM BSA-Palmitate Saturated Fatty Acid Complex (BioSite cat: 154–29558), 100, 200, or 400 μM BSA Control for BSA-Fatty Acid Complexes (BioSite cat: 154–29556), 0.5 mM H_2_O_2_ (Sigma–Aldrich cat: 216763), 40 ng/mL IL-6 (R&D systems cat: 206-IL), 10 ng/mL IFN-γ (cat: PeproTech cat: 315–05), or vehicles. Unless otherwise stated in the figure legends, experiments were conducted using the concentrations and time specified above.

### 2.16 Sox4 and Smad4 knockdown via siRNA

Primary mouse hepatocytes were used for siRNA-mediated knockdown experiments. Cells were isolated and transfected the day after with 2.8 μM siSox4, siSmad4, or siCtrl using Viromer Blue transfection reagent (Lipocalyx cat: VB-01LB-00). Viromer Blue (1 μL/transfection) was dissolved in Blue buffer and mixed with siRNAs followed by 15 min incubation at room temperature before adding the transfection mix to cells. 24 h post transfection, cells were treated with 10 ng/mL TGF-β1 and incubated for additional 24 h before cell harvest and RNA analysis.

### 2.17 Zfp697 knockdown via AdshRNA

AdshZfp697 construct: Ad (RGD)-GFP-U6-m-Zfp697-shRNA from Vector Biolabs (SKU# shADV-276960), with EGFP tag under CMV promoter. ShRNA sequence: 5′-CACC GGAATGGGCTCTAATCCATGGCTCGAG CCATGGATTAGAGCCCATTCC TTTTT-3'. Primary mouse hepatocytes were infected with MOI 50 immediately after seeding. RNA was harvested 53 h post-infection. Huh7 cells were seeded in 12-well plates and cultured until 60% confluency, then infected with MOI 100 in standard growth media without P/S and with reduced FBS (2%). The media changed to regular growth media 6 h post-infection. 48 h after infection, 10 ng/ml TGF-β1 or diluent was added and cells were harvested for RNA extraction at 1, 4, 8, 24, and 48 h post-treatment or protein extraction 1 h post-treatment.

### 2.18 Zfp697 overexpression via AdZfp697

Recombinant adenovirus expressing FLAG-Zfp697 was generated as previously described in Correia et al., 2024 [10]. Primary mouse hepatocytes were seeded and infected immediately with infected with AdZfp697, or control AdGFP, at MOI 100. Two hours after seeding, cells were washed once in PBS and media was replaced by maintenance media. At 24 h post-infection, hepatocytes were stimulated with 10 ng/mL TGF-β1 or vehicle and incubated for an additional 24 h. Cells were then harvested for RNA isolation and gene-expression analysis. Huh7 cells were treated with AdZfp697, or control AdGFP, for 48 h followed by 1 h treatment with 10 ng/mL TGF-β1 or vehicle. Cells were then harvested for protein extraction as described below.

### 2.19 Protein extraction and immunoblotting

Huh7 cells were washed once in ice-cold PBS and scraped into 1 mL PBS supplemented with protease and phosphates inhibitors (cOmplete™ EDTA-Free Protease Inhibitor Cocktail, Roche cat: 11873580001; PhosSTOP™, Roche cat: 4906845001) on ice. Cells were pelleted by centrifugation (500 x *g*, 5 min, 4 °C), the supernatant was discarded, and pellets were resuspended in protein lysis buffer containing 50 mM Tris–Hcl (pH 7.4), 180 mM NaCl, 1 mM EDTA, 1% Triton X-100, and 20% glycerol, supplemented before use with protease and phosphatase inhibitors and 1 mM dithiothreitol (DTT). Lysates were incubated on ice for 15 min with intermittent vortexing and subsequently sonicated using a Diagenode Bioruptor® (5 cycles of 30 s on high power and 30 s off at 4 °C). Cellular debris were removed by centrifugation (18,000 x *g*, 20 min, 4 °C), and clarified lysates transferred to fresh tubes. Protein concentrations were determined using the Bradford assay.

Protein extracts were diluted in lysis buffer and 4x Laemmli Sample Buffer (Bio-Rad cat: 1610747) supplemented with 2-mercaptoethanol and heated for 10 min at 70 °C. Equal amounts of protein (15 μg) were separated on 4–20% Mini-PROTEAN® TGX™ precast gels (Bio-Rad) and transferred to polyvinylidene difluoride (PVDF) membranes previously activated in EtOH (80 mA, 24 h, 4 °C). Total protein loading and transfer efficiency were assessed using the No-Stain™ Protein Labeling Reagent (Invitrogen cat: A44449) according to manufacturer's instructions.

Membranes were blocked for 1 h in 5% BSA in Tris-buffered saline containing 0.1% Tween-20 (TBST) at room temperature and incubated overnight (16 h, 4 °C) with anti-phospho-Smad2 (Ser465/467) antibody (1:1,000 dilution in 5% BSA/TBST, Millipore cat: 04–953). After washing with TBST, membranes were incubated for 1 h at room temperature with horseradish peroxidase (HRP)-conjugated goat anti-rabbit IgG (1:10,000 dilution in 5% BSA/TBST, Abcam cat: ab6721). Following washing with TBST, protein bands were visualized using enhanced chemiluminescence (Amersham ECL Prime; Cytiva cat: 89168–782) and imaged with an iBright™ CL1500 system.

Band intensities and total protein lane intensities were quantified using iBright analysis software. Phospho-Smad2 signal intensities were normalized to the corresponding total protein signal of the same lane (No-Stain™) and subsequently normalized to the mean value of the respective control group (AdshCtrl or AdGFP) within each gel.

### 2.20 RNA isolation

RNA was obtained from liver tissue or cell samples using standard phenol-chloroform extraction. RNA was precipitated in isopropanol, pellets were washed in 70% EtOH, and dissolved in nuclease-free water. RNA concentrations and purity were determined using a NanoDrop ND-1000.

### 2.21 RT-qPCR

RNA was DNase-treated (Invitrogen cat: 18,068–015) in reaction buffer (Invitrogen cat: Y02340) for 15 min, followed by DNase-inactivation with the addition of EDTA and incubation at 65 °C for 10 min. Reverse transcription was performed using the High-Capacity RNA-to-cDNA™ Kit (Applied Biosystems cat: 4387406). qPCR was performed using ViiA7 or QuantStudio 6 real-time PCR systems (Applied Biosystems) and Power SYBR Green PCR Master Mix (Applied Biosystems cat: 4368708). Primers are listed in Table S5.

### 2.22 3′-tag RNA-sequencing analysis

Total RNA was extracted using the Direct-zol™ RNA Miniprep Kit (Zymo Research) and submitted for 3′-end counting RNA-seq library preparation and Illumina sequencing. Raw reads were quality-filtered with FastP (v0.24.0), aligned to GRCm39 using STAR (v2.7.11 b), and deduplicated using UMICollapse (v1.1.0). Gene-level counts were quantified with featureCounts (Subread v2.1.1). Differential expression analysis was restricted to protein-coding genes and performed using edgeR (Bioconductor 3.20) in R (v4.4.2), with TMM normalization and a quasi-likelihood framework. Pairwise contrasts assessed diet and genotype effects within a 2 × 2 factorial design (Flox/HepKO × chow/MASH), and genes with FDR <0.05 and |log2FC| ≥ 1 were considered differentially expressed. Gene set enrichment was performed using clusterProfiler, with ORA for up- and downregulated gene lists and GSEA (fgsea algorithm) using a sign (log2FC) × −log10 (p-value) ranking, against MSigDB Hallmark and KEGG databases. Disease-gene associations were identified via Metascape [18] using the DisGeNET database. All enrichment results were corrected using the Benjamini–Hochberg method (FDR <0.05).

### 2.23 eCLIP sequencing

Livers were collected from 10-week-old male C57BL/6J mice injected with Ad-FLAG-Zfp697 (n = 3) one-week post-termination and pulverized on dry ice. The resulting liver powder was thinly spread on a petri dish and UV-crosslinked at 254 nm with 400 mJ/cm^2^ using a Stratagene Stratalinker UV 1800, keeping samples frozen throughout. Crosslinked samples were stored at −80 °C until processing. The eCLIP protocol was performed as described in Blue et al., 2022 [19] and Nostrand et al., 2016 [20]. Briefly, cells were lysed in lysis buffer (50 mM HEPES pH 7.4, 150 mM NaCl, 0.5% NP-40, 0.1% SDS, 0.5 mM DTT, 1 mM PMSF, and protease inhibitors). RNA was partially digested using RNase I (Thermo Fisher Scientific cat: AM2295). Lysates were centrifuged at maximum speed and the supernatant was subjected to immunoprecipitation with FLAG antibody (Sigma–Aldrich cat: F3165) conjugated to protein A/G magnetic beads. Input samples were saved prior to washing. Next, the RNA-protein complexes were washed with high salt wash buffer (50 mM HEPES pH 7.4, 1 M NaCl, 1 mM EDTA, 0.1% SDS, 1% NP-40), low salt wash buffer (50 mM HEPES pH 7.4, 150 mM NaCl, 1 mM EDTA, 0.1% SDS, 1% NP-40), and PNK buffer (50 mM Tris pH 7.4, 10 mM MgCl_2_, 0.5% NP-40). RNA-protein complexes were ligated to 3′ adapters, reverse transcribed using SuperScript III (Thermo Fisher Scientific cat: 12574026), and cDNA was purified with Agencourt AMPure XP beads (Beckman Coulter). Libraries were PCR amplified using Q5 High-Fidelity DNA Polymerase (NEB cat: M0541) with barcoded primers, purified again with AMPure XP beads, and sequenced on an Illumina NovaSeq platform.

### 2.24 Interacting sites, motif analysis, and GO-term analysis

Raw eCLIP fastq files were processed using Skipper [21] as described in Xu et al., 2024 [22], using the mm10 genome and GENCODE vM10 annotations. De novo motif analysis was performed with HOMER on Skipper-identified reproducible enriched 75-nucleotide windows centered on local maxima, yielding significantly enriched sequence motifs. Examples of interaction sites were visualized in IGV (version 2.19.4) [23], [24]. GO Biological Process enrichment among Zfp697-bound RNAs was assessed using Enricher [25], [26], [27]. The input samples were used as the background list and RNAs were included if they had at least 10 reads in at least 2 of the 3 input files.

### 2.25 GST pull-down

Full-length amplified Zfp697 was cloned into pJet cloning vector and subcloned in pGEX-4T3 in frame with GST. E. coli. BL21 cells were transformed with the constructs. Bacterial culture media was supplemented with 50 μM ZnCl_2_ to support Zfp697 protein folding. Protein expression was induced by addition of 100 μM IPTG. GST-Zfp697 was purified by using glutathione sepharose 4 B beads (GE healthcare) as described below.

Mouse liver nuclear extracts were prepared as described in the following. 2-Month-old C57BL/6 N male mice were euthanatized with isoflurane before livers were perfused through vena cava with perfusion buffer (HBSS (Gibco cat: 14175–053) containing 21.4 mM NaHCO_3_ and 684.4 μM EDTA) followed by liver digest media (Gibco cat: 17703034). Livers were ruptured, filtered through 70 μm cell strainer, pooled and placed on ice. The liver cells were then washed 2 times with cold PBS centrifuging 10 min at 2,500 rpm 4 °C before cell pellet was resuspended and homogenized by repeated pipetting in cold hypotonic buffer (10 mM Tris–HCl pH 7.4, 10 mM KCl, and 1.5 mM MgCl_2_) containing protease and phosphatase inhibitors. The sample was incubated 10 min and further homogenized using a douncer. Cell lysate was pelleted by centrifugation at 3,900 rpm for 15 min 4 °C and resuspend in cold low salt buffer (20 mM Tris–HCl pH 7.4, 2.5% glycerol, 1.5 mM MgCl_2_, 2 mM EDTA, and 20 mM KCl) containing protease and phosphatase inhibitors. At continuous shaking high salt buffer containing the same content as the low salt buffer but with 1.2 M KCl was added to the sample and incubated at 4 °C for 30 min with rotation. Sample was centrifuged at 14,000 rpm for 30 min 4 °C and the supernatant was transferred to a new tube. The conductivity was measured using a HI-8333 conductivity meter from Hanna Instruments and BC-0 without EDTA was added to adjust the conductivity to 8.6 mS/cm lowering the final EDTA concentration to 0.9 mM minimizing the chelation of zinc ions important for Zfp697 protein folding during GST-pull down. NP-40 was added to a final concentration of 0.05% and the sample was snap-frozen in liquid N_2_ and stored at −80 °C.

GST and GST-Zfp697 were incubated with glutathione sepharose 4 B beads previously equilibrated in BC-500 without EDTA, in BC-100 without EDTA including DTT, PMSF, and 0.1% NP-40 overnight at 4 °C. GST-beads were washed 5 times in BC-180 containing DTT, PMSF, and 0.1% NP-40 followed by 2 × 20 min washes in the same buffer containing 0.2% sarkosyl. After additional 3 washes in BC-180 containing DTT, PMSF, and 0.1% NP-40 samples were blocked with 1 μg/mL BSA in BC-180 containing DTT, PMSF, and 0.05% NP-40 for 1 h at 4 °C. GST-pull down was then performed incubating the GST-beads with 2 mg liver nuclear extracts in BC-180 containing DTT, PMSF, and 0.05% NP-40 overnight at 4 °C. Beads were washed 2 times in BC-180 containing DTT, PMSF, and 0.05% NP-40 and 2 times in buffer (20 mM Tris–HCl pH 7.4, 2 mM EDTA, and 180 mM KCl). After resuspending the beads for the last wash 10% was saved for silver-staining while supernatant was removed from the remaining 90% sample and beads were stored at −80 °C until LC-MS analysis.

Silver-staining: Beads were resuspended in 2*x* laemmli buffer and incubated at 80 °C for 10 min, centrifuged at top speed for 1 min at room temperature, and loaded into a 4–15% Novex tris-glycine gel. Silver staining was performed using Invitrogen's Silver Quest Silver staining kit and finally dried the gel using Invitrogen's Gel-Dry Drying System.

### 2.26 LC-MS/MS: on-bead digestion

Following IP washes, bead bound protein complexes were digested directly on beads by addition of 0.75 μg (1 μg/μL) of trypsin (sequencing grade, Promega) in 50 mM NaHCO_3_ buffer. Beads were gently tapped to ensure even suspension of trypsin solution and incubated at 37 °C with mild agitation for 2 h. Beads were then vortexed to ensure the complex falls off the beads, and the partially digested complex was transferred into clean tubes to separate it from the beads and incubated at 37 °C for 16 h without agitation. Resulting peptides were extracted into LC-MS vials by 2.5% formic acid (FA) in 50% acetonitrile (ACN) and 100% ACN with addition of polyethylene glycol (20,000; final concentration 0.001%) [28] and concentrated in a SpeedVac concentrator (Thermo Fisher Scientific).

### 2.27 LC-MS/MS: analysis of tryptic digests

LC-MS/MS analyses of all peptide mixtures were done using RSLCnano system connected to Orbitrap Fusion Lumos mass spectrometer (Thermo Fisher Scientific). Prior to LC separation, tryptic digests were online concentrated and desalted using trapping column (100 μm × 30 mm, column compartment temperature of 40 °C) filled with 3.5-μm X-Bridge BEH 130C18 sorbent (Waters). After washing of trapping column with 0.1% FA, the peptides were eluted (flow rate - 300 nL/min) from the trapping column onto an analytical column (Acclaim Pepmap100C18, 3 μm particles, 75 μm × 500 mm; column compartment temperature of 40 °C, Thermo Fisher Scientific) by 100 min nonlinear gradient program (0 min: 1%, 70 min: 30%, 100 min: 56% of mobile phase B; mobile phase A: 0.1% FA in water; mobile phase B: 0.1% FA in 80% ACN). Equilibration of the trapping column and the analytical column was done prior to sample injection to sample loop. The analytical column outlet was directly connected to the Digital PicoView 550 (New Objective) ion source with sheath gas option and SilicaTip emitter (New Objective; FS360-20-15-N-20-C12) utilization. ABIRD (Active Background Ion Reduction Device, ESI Source Solutions) was installed.

MS data were acquired on Orbitrap Fusion Lumos mass spectrometer in a data-dependent strategy with cycle time for 3 s and with survey scan (350–2000 *m*/*z*). The resolution of the survey scan was 60,000 (*m*/*z* 200) with a target value of 4 × 10^5^ ions and maximum injection time of 50 ms. HCD MS/MS (30% relative fragmentation energy, normal mass range) spectra were acquired with a target value of 5.0 × 10^4^ and resolution of 30,000 (*m*/*z* 200). The maximum injection time for MS/MS was 54 ms. Dynamic exclusion was enabled for 60 s after one MS/MS spectra acquisition. The isolation window for MS/MS fragmentation was set to 1.6 *m*/*z*.

The analysis of the mass spectrometric RAW data files was carried out using the MaxQuant software (version 1.6.0.16) using default settings unless otherwise noted. MS/MS ion searches were done against modified cRAP database (based on http://www.thegpm.org/crap) containing protein contaminants like keratin, trypsin etc., and UniProtKB protein database for Mus musculus (https://ftp.uniprot.org/pub/databases/uniprot/current_release/knowledgebase/reference_proteomes/Eukaryota/UP000000589/UP000000589_10,090. fasta.gz; downloaded 2017-12-20, version 2017/12, number of protein sequences: 22,276). Oxidation of methionine, deamidation (N, Q) and acetylation (protein N-terminus) as optional modification, and trypsin/P enzyme with 2 allowed missed cleavages were set. Peptides and proteins with FDR threshold <0.01 and proteins having at least one unique or razor peptide were considered only. Match between runs was set among all analyzed samples. Protein abundance was assessed using protein intensities calculated by MaxQuant.

Protein intensities reported in proteinGroups.txt file (output of MaxQuant) were further processed using the software container environment (https://github.com/OmicsWorkflows), version 3.7.2a. Processing workflow is available upon request. Briefly, it covered: a) removal of decoy hits and contaminant protein groups, b) protein group intensities log_2_ transformation, c) median normalization, d) differential expression using LIMMA statistical test with Benjamini-Hochberg adjustment of the resulting P values. Proteins passing the threshold of log_2_FC > 1 and adjusted p-value<0.05 in addition to those proteins solely detected in the GST-Zfp697 samples were considered for further evaluations.

Proteins were categorized based on predefined fold-change thresholds, and clustering analysis confirmed sample replicate consistency. Protein–protein interactions were analyzed using MiST [29] and SAINT [30], filtering and scoring the interactions which were subsequently visualized using STRING network analysis [31] and functional nodes and protein complexes within the networks were identified with ClusterOne [32] and subsequently visualized using CytoScape [33].

### 2.28 Quantification and statistical analysis

Data analysis was performed using Excel, GraphPad Prism, and CalR [34]. Statistical analysis and sample sizes are found in the figure legends.

### 2.29 Publicly available data

As described in the main text and figure legends, the following publicly available data was examined and incorporated into this manuscript. RNA-seq data from male C57BL/6 mice treated with 400 mg/L thioacetamide in drinking water for 16 weeks to induce liver fibrosis (GEO: GSE216326) [35]. RNA-seq data from male C57BL/6J mice with acute acetaminophen-induced (350 mg/kg) liver injury. Expression data from 12, 24, 36, 48, and 72 h post injury and one control group (n = 4) (GEO: GSE111828) [36]. Single-nuclei RNA-seq data, hepatocyte population, from male and female mice treated with a single dose of acetaminophen (300 mg/kg (males) or 350 mg/kg (females) to induce liver injury. Expression data from 0, 3, 6, 12, 18, 24, 30, 36, 42, 48, 72, 120, 168, and 336 h post injury (n = 3) from Liver Regeneration Atlas (https://shiny.igc.ed.ac.uk/7efa5350ba94425388e47ea7cdd5aa64/) (GEO: GSE223561) [37]. DNA microarray data from patients with alcoholic hepatitis (n = 15) and healthy controls (n = 7) (GEO: GSE28619) [38]. RNA-seq data from HSCs isolated from CCl_4_-treated mice (GEO: GSE134512) [39]. RNA-seq data from human liver tissue adjacent to cancer from 56 patients with hepatocellular carcinoma (chronic liver disease) and liver tissue from 13 patients without hepatocellular carcinoma as controls. Mixed males and females (N = 69) (GEO: GSE244826) [40]. SMAD2, SMAD3, SMAD4, and SOX4 ChIP-seq tracks from human cell lines and mouse tissues and cell lines were downloaded from ChIP-Atlas (https://chip-atlas.org/) [41], [42], [43], [44] and visualized in IGV (version 2.19.4) [23], [24] mm10 and hg38. Primary mouse myotubes eCLIP data [10]. Single-cell data from mouse liver [45] available at livercellatlas.org.

## 3 Results

### 3.1 Liver *Zfp697/ZNF697* expression is associated with hepatic metabolic disease and injury

We have recently shown that the transient increase in *Zfp697* expression in skeletal muscle is necessary for the tissue to recover from atrophy and injury. Conversely, *Zfp697* expression has been shown to be chronically high in skeletal muscle disease, kidney injury, and heart failure and dysfunction [10]. Together, these findings led us to investigate Zfp697 levels in liver disease and injury where unresolved inflammation and extracellular matrix (ECM) remodeling are strong components.

We started by measuring liver *Zfp6*97 mRNA expression in three different mouse models of liver disease or injury. First, we challenged mice with a methionine- and choline-deficient (MCD) diet, a commonly used dietary model of MASH [46], [47]. As expected, after 7 days on this diet mice displayed lower body and liver weight as compared to chow-fed control mice (Fig. S1A–D), in addition to a hepatic transcriptomic profile indicating inflammation, tissue injury response, and extracellular matrix remodeling (Fig. S1E) [47], [48]. In livers from MCD-fed mice, *Zfp6*97 mRNA expression was significantly higher when compared to controls (Fig. 1A). Similarly, we found a 2-fold increase in hepatic *Zfp697* expression in mice fed a fast food (FF) diet for 25 weeks, using RNA samples from a previously published, well-characterized FF model [49] (Fig. 1B), and a 10-fold increase in livers of mice 16 h after administration of the hepatotoxic chemical carbon tetrachloride (CCl_4_) (Fig. 1C). Like the MCD model, both the FF and CCl_4_ mouse models exhibited elevated expression of genes associated with inflammation, injury response, and extracellular matrix remodeling as expected [48], [50] consistent with *Zfp697* upregulation in a context of hepatic inflammation and tissue remodeling (Fig. S1F–G). In line with these results, analysis of publicly available data showed higher expression of *Zfp697* in livers of mice with thioacetamide (TAA)-induced fibrosis (Fig. 1D) (GEO: GSE216326) [35], and transiently elevated *Zfp697* expression after acute acetaminophen-induced liver injury as seen both in bulk liver samples (Fig. 1E) (GEO: GSE111828) [36] and in the hepatocyte population in single-cell data (Fig. 1F) (GEO: GSE223561) [37].

**Fig. 1:**
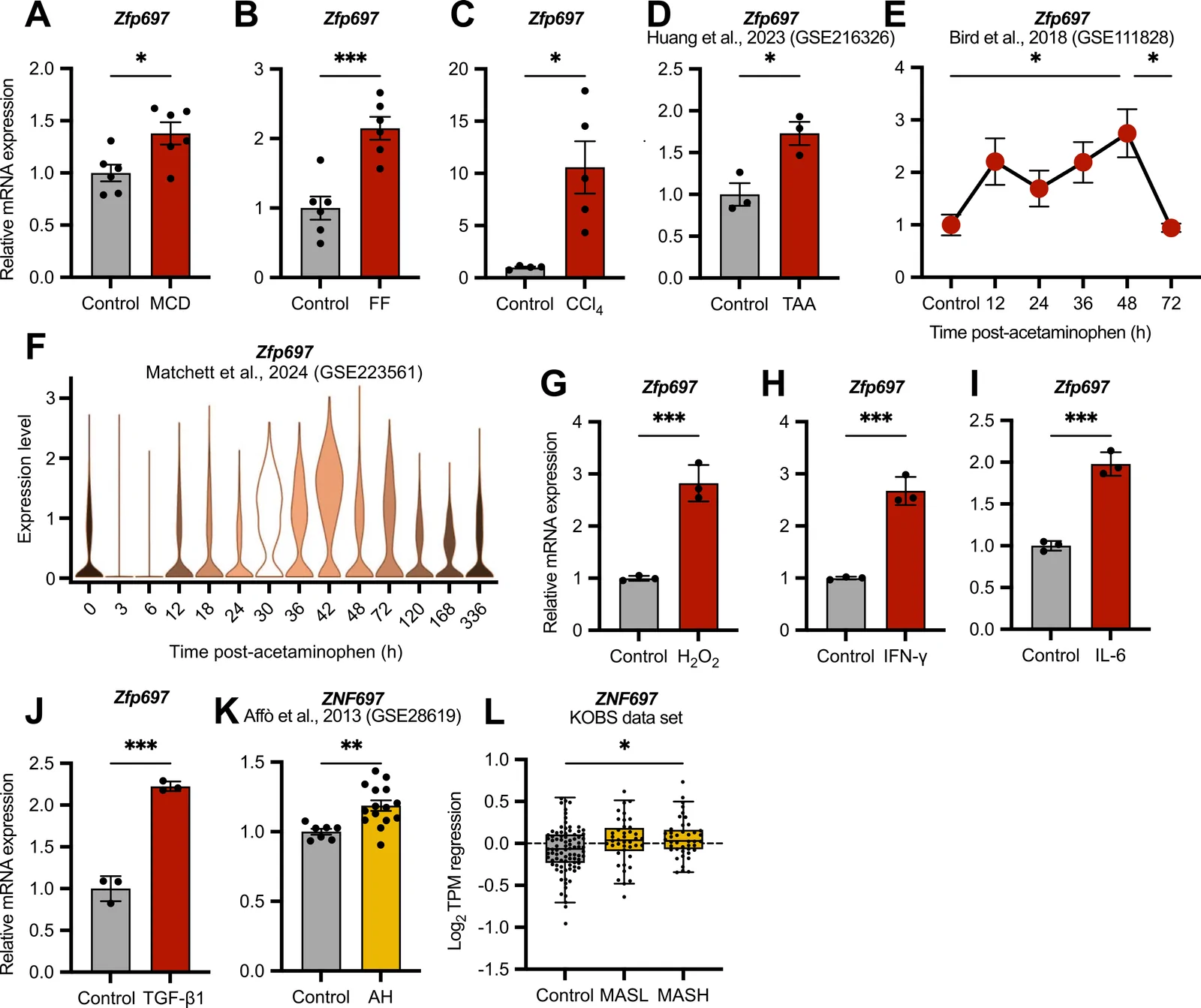
**Liver *Zfp697* expression in hepatic and metabolic diseases.***Zfp6*97 mRNA expression in livers of mice: (**A**) fed a methionine- and choline-deficient (MCD) diet for 7 days (n = 6), (**B**) fed a fast food (FF) diet for 25 weeks (n = 6), (**C**) 16 h post carbon tetrachloride (CCl_4_) injury (n = 4–5), (**D**) treated for 16 weeks with thioacetamide (TAA) (GEO: GSE216326) (n = 3), and (**E**) treated with a single dose of acetaminophen (GEO: GSE111828) (n = 4). (**F**) *Zfp697* expression in the hepatocyte population in livers of mice treated with a single dose of acetaminophen (single-nuclei RNA-seq data) (GEO: GSE223561). (**G**) *Zfp6*97 mRNA expression in primary mouse hepatocytes after 24 h: G) under oxidative stress (0.5 mM H_2_O_2_) (n = 3), treated with (**H**) 10 ng/mL IFN-γ (n = 3), (**I**) 40 ng/mL IL-6 (n = 3), and (**J**) 10 ng/mL TGF-β1 (n = 3). (**K**) *ZNF69*7 mRNA expression in livers from patients with alcoholic hepatitis (AH) (n = 15) or healthy controls (n = 7) (GEO: GSE28619). (**L**) *ZNF697* expression in livers from patients with different degrees of MASLD (KOBS). Statistics: (A-D, G-K) Student's unpaired t-test, (E) One-way ANOVA with Tukey's test with correction for multiple comparisons, (L) Kruskal–Wallis test (p = 0.015), followed by pairwise Wilcoxon rank-sum tests with Benjamini–Hochberg adjustment. ∗p<0.05, ∗∗p<0.01, ∗∗∗p<0.001. Error bars represent (A-E, K) SEM, (G–J) SD.

Liver disease and injury are associated with an increase in oxidative stress and elevated inflammation markers such as IFN-γ, IL-6, and TGF-β1 [3], [51], [52], [53]. To test if any of these stimuli could regulate *Zfp697* expression, we treated mouse primary hepatocytes with the oxidative stress agent H_2_O_2_ or different cytokines. We observed an increase in *Zfp697* with all the tested conditions, albeit to varying levels (Fig. 1G-J). To verify that each treatment worked as expected, we measured some of the corresponding classical downstream response genes (Fig. S1H–J).

The association between increased *Zfp697* expression and liver disease and injury could also be seen in human patients. Analysis of publicly available datasets showed that *ZNF697* is higher in livers of patients with alcoholic hepatitis as compared to control liver samples from non-diseased donors (GEO: GSE28619) [38] (Fig. 1K). In addition, analysis of liver biopsies from the Kuopio Obesity Surgery Study (KOBS) revealed a significant overall difference in *ZNF697* expression across MASLD stages. *ZNF697* expression was higher in livers from patients with MASH compared with healthy controls (Fig. 1L).

Alongside hepatocytes, hepatic stellate cells (HSCs) play a key role in liver disease [54]. We therefore analyzed publicly available data from HSCs isolated from CCl_4_-treated mice (GEO: GSE134512) [39] (Fig. S1K), where we could also determine a higher *Zfp697* expression compared to controls. Furthermore, we treated the human HSC cell line, LX-2, with palmitate and TGF-β1, to mimic lipotoxic and profibrotic signaling associated with liver injury [55], [56], which also resulted in higher *ZNF697* expression (Fig. S1L-M).

Thus, *Zfp697* and *ZNF697* transcripts are more abundant in mouse and human liver disease and injury, with potential contributions from both hepatocytes and HSCs.

### 3.2 Zfp697 expression level dictates hepatic inflammation marker gene expression

Based on the finding that *Zfp697/ZNF697* expression is associated with liver disease and injury, we next asked if Zfp697 is sufficient and/or necessary to drive disease-responsive transcriptional changes. To test this, we performed Zfp697 gain- and loss-of-function in mouse primary hepatocytes using adenoviral-mediated expression and shRNA knockdown, respectively. Indeed, ectopic *Zfp697* expression in primary hepatocytes (Fig. S2A) was sufficient to increase the expression of the pro-inflammatory and immune-related genes *Ccl2*, *Ccl7*, *Ccl9*, *Ccl20, Cd14*, and *Cxcl11* (Fig. S2B), as well as the genes *Acta2* and *Plaur,* involved in ECM remodeling (Fig. S2C). Interestingly, this Zfp697 gain-of-function condition was also associated with reduced expression of genes related to the TGF-β signaling pathway: *Tgfb2*, *Tgfbr3*, and *Sox4* (Fig. S2D). Conversely, shRNA-mediated knockdown of Zfp697 led to altered expression of genes in the same pathway, with higher, or trending higher, expression of *Tgfb1* and *Tgfbr1* and lower expression of *Smad7* and *Traf6* (Fig. S2E–H). Thus, Zfp697 is an active modulator of several disease- and injury-related gene programs in hepatocytes. Modulating Zfp697 expression alters the expression of genes related to inflammation, chemotaxis, ECM remodeling, and components of the TGF-β signaling pathway. The transcriptional responses were not uniformly reciprocal between gain- and loss-of-function experiments, suggesting that Zfp697 regulates these pathways in a context-dependent manner.

### 3.3 *Zfp697*/*ZNF697* expression correlates with liver disease marker genes

Liver disease and injury are associated with extensive adaptation of the hepatic transcriptome [57], [58], [59]. To gain insight into Zfp697 mRNA expression in relation to the disease response, we sought to determine whether *Zfp697* expression correlates with disease- and injury-associated genes presented in Fig. S1E–G. Indeed, in MCD diet-fed mice, we found a strong correlation between *Zfp697* expression and markers of inflammation and immune cell infiltration (*Cd68*, *Cd11b*, *Cd14*, and *Ccl2*) and ECM remodeling (*Tgfb1, Timp1*), two hallmark features of the hepatic response to injury (Fig. 2A). Further expanding the analysis to a broader gene panel and screening across disease and injury mouse models; MCD diet, FF diet, and CCl_4_-injected mice, we found strong correlations between *Zfp697* expression levels and those of genes associated with inflammation and immune response, stress and damage response, ECM remodeling, cell cycle and proliferation, and metabolism (Fig. 2B). This was seen in a model-specific manner, reflecting the differences between chronic and acute liver injury response. For example, expression of *F4/80*, a macrophage marker, was specific to the diet-induced models, consistent with its reported upregulation during later stages of liver injury [60]. Similarly, *Timp2* and *Mmp2*, established fibrosis markers, were expressed at higher levels only in the chronic models, reflecting their association with later phases of injury and fibrogenesis, which are absent in the acute CCl_4_ setting [61], [62], [63]. Importantly, the general correlations between *Zfp697* and disease marker genes were conserved in humans, as seen in RNA-seq data from patients with chronic liver disease and controls (GEO: GSE244826) [40]. Here, we observed strong correlations between *ZNF697* and the classical liver disease-marker genes: *TGFB1*, *TGFBR2*, *SMAD4*, *CD163*, *NFKB1*, and *FN1* (Fig. 2C), in addition to several others related to inflammation, ECM remodeling, and apoptosis (Fig. 2D). These findings were further supported by analysis of the independent KOBS dataset (Table S1), demonstrating consistent correlations between *ZNF697* and liver disease-marker genes.

**Fig. 2:**
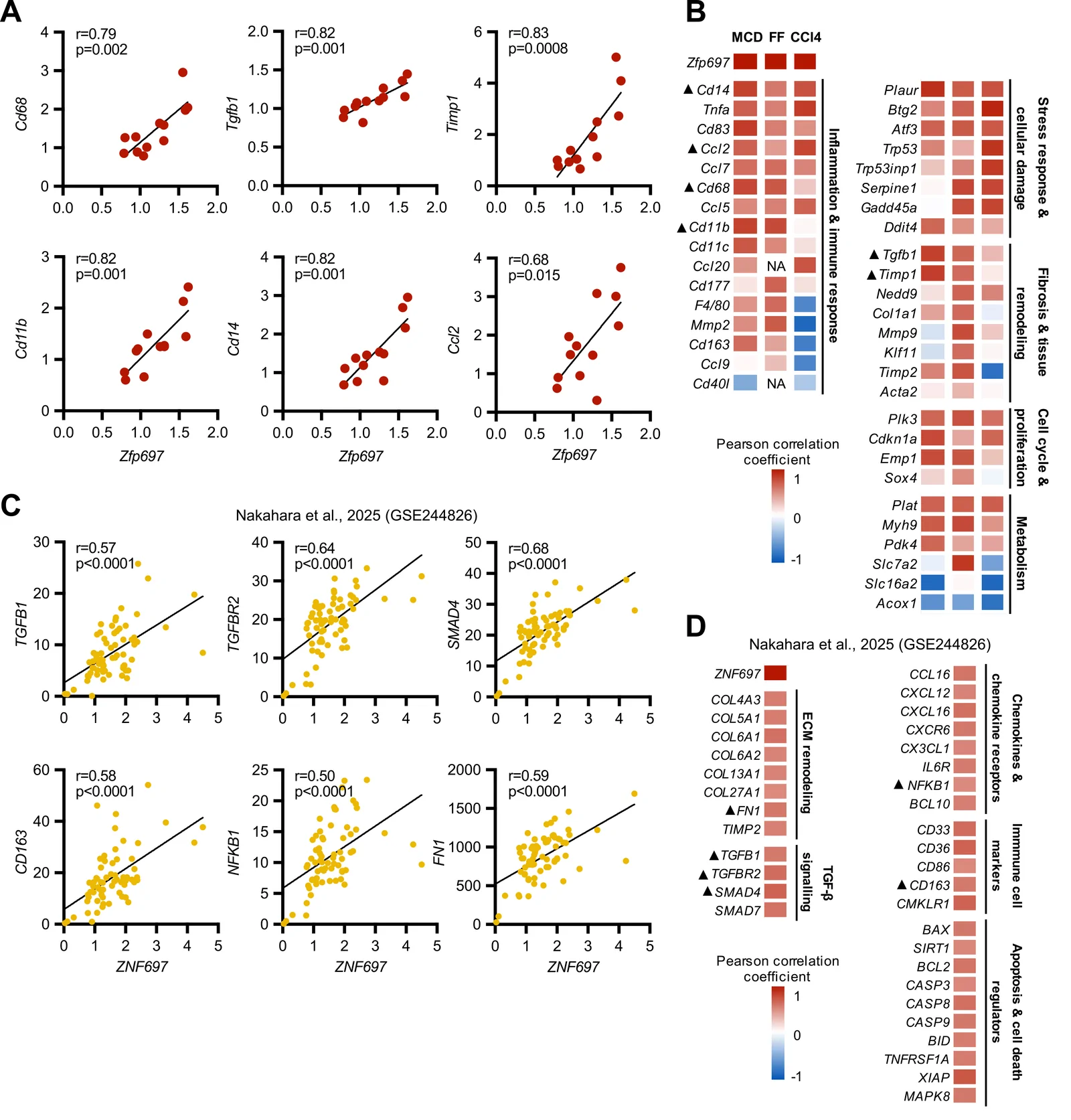
***Zfp697/ZNF697* expression correlates with inflammation, ECM remodeling, and stress response genes.** (**A**) Correlation plots showing fold change expression of *Zfp697* and *Cd68*, *Tgfb1*, *Timp1*, *Cd11b*, *Cd14*, and *Ccl2* in methionine- and choline-deficient (MCD) diet-fed mice compared to chow-fed controls (N = 12). (**B**) Heatmap of Pearson correlation coefficients between *Zfp697* and genes related to inflammation, stress, tissue remodeling, cell cycle, and metabolism across three models of liver disease and injury: MCD diet, fast food (FF) diet, and carbon tetrachloride (CCl_4_) injury. Black triangles indicate correlations in the MCD model also shown in panel A. (**C**) Correlation plots of *ZNF697* with *TGFB1, TGFBR2, SMAD4, CD163, NFKB1*, and *FN1* (TPM-normalized read counts) in human liver biopsies (Nakahara et al., 2025 (GEO: GSE244826)) (N = 69). (**D**) Heatmap showing *ZNF697* correlations with genes involved in extracellular matrix (ECM) remodeling, TGF-β signaling, chemokine signaling, immune cell markers, and cell death. Black triangles denote overlaps with panel C. Statistics: Pearson correlation coefficients (r) and t-test for the significance of the regression slopes (p).

### 3.4 Hepatic TGF-β1 signaling regulates *Zfp697* expression

To identify liver disease- and injury-induced cues regulating *Zfp697* expression, we examined classical signaling pathways associated with these conditions. In addition to the results obtained with mouse primary hepatocytes shown in Fig. 1J, we also found that ZNF697 mRNA expression was higher in TGF-β1-treated human primary hepatocytes (Fig. 3A), and in the human hepatocyte cell line Huh7, where its regulation was comparable to that of classical TGF-β1 target genes (Fig. S3A–B). Time-course experiments further confirmed a progressive increase in *Zfp697/ZNF697* expression following TGF-β1 stimulation in both mouse primary hepatocytes and Huh7 cells (Fig. 3B, Fig. S3C). Given the conserved and sustained response across these systems, we focused on TGF-β1 signaling for further investigation.

**Figure 3:**
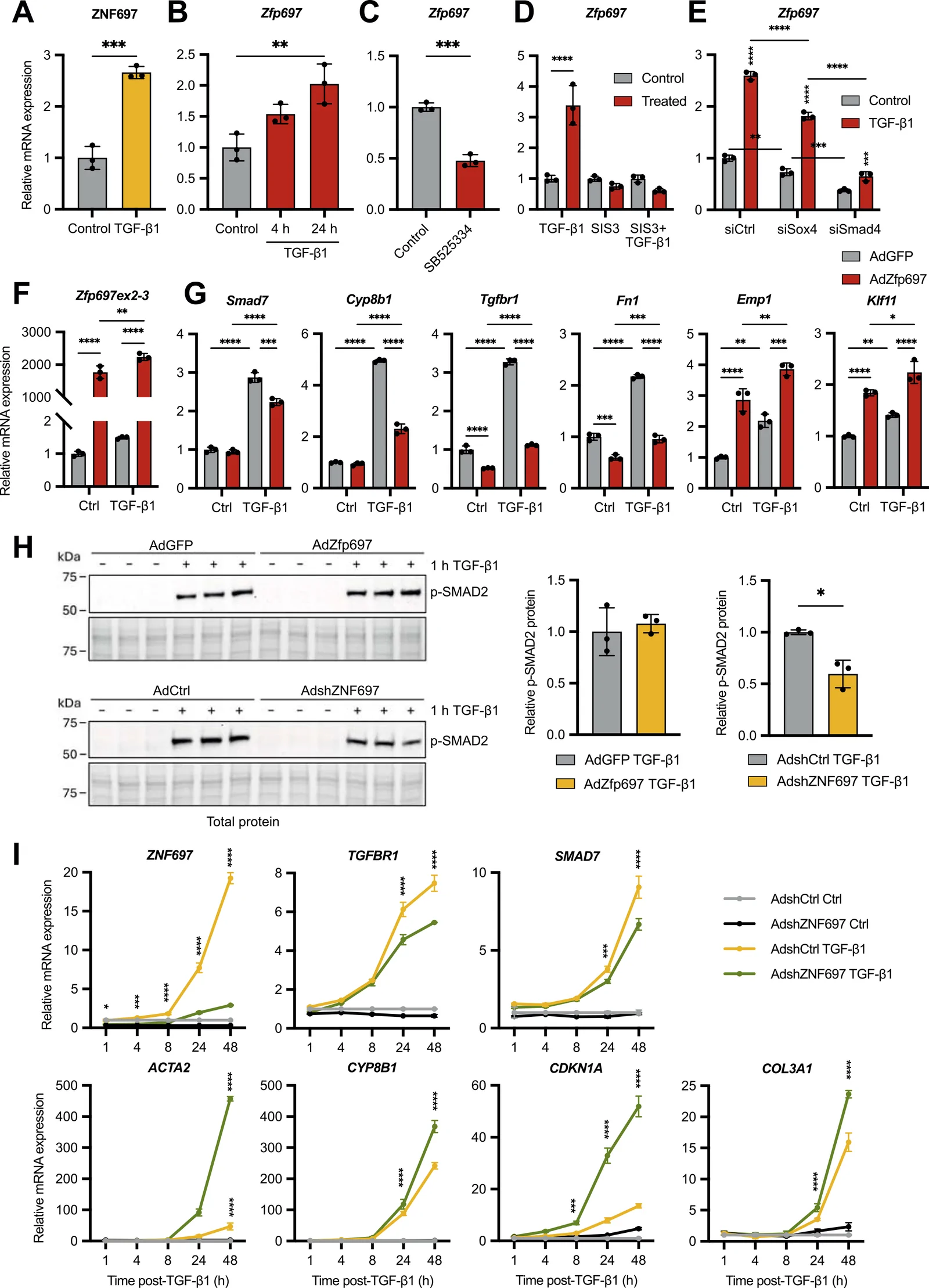
**Bidirectional regulation of hepatic Zfp697 and TGF-β1 signaling.** (**A**) ZNF697 mRNA expression in primary human hepatocytes treated with 10 ng/mL TGF-β1 for 24 h (n = 3). B-E) Zfp697 mRNA expression in primary mouse hepatocytes treated with (**B**) 10 ng/mL TGF-β1 for 4 h and 24 h (n = 3), (**C**) 5 μM TGFBR1 (ALK5) inhibitor SB525334 for 24 h (n = 3), (**D**) 10 ng/mL TGF-β1, 10 μM SIS3, and their combination with 30 min pre-treatment with SIS3 for 24 h (n = 3), and (**E**) transfected with siSox4, siSmad4, or siControl for 24 h followed by 10 ng/mL TGF-β1 for 24 h (n = 3). (**F**) mRNA expression of *Zfp697* and (**G**) TGF-β1 target genes in primary mouse hepatocytes pre-treated with AdZfp697 or AdGFP for 24 h, followed by 10 ng/mL TGF-β1 for 24 h (n = 3). (**H**) p-SMAD2 levels in Huh7 treated with AdZfp697, AdshZNF697, or controls for 48 h followed by 1 h treatment with 10 ng/mL TGF-β1. PSMAD2 signal intensities were normalized to the corresponding total protein signal of the same lane and subsequently normalized to the mean value of the respective control group (AdshCtrl or AdGFP) within each gel. Quantification includes only TGF-β1-treated samples (**I**) mRNA expression of *ZNF697* and TGF-β1 target genes in Huh7 cells treated with AdshZNF697 or AdshCtrl for 48 h, followed by 10 ng/mL TGF-β1 treatment for 1, 4, 8, 24, and 48 h (n = 3). Data shown as relative to AdshCtrl Ctrl. Statistics: (A, C) Student's unpaired t-test, (B) One-way ANOVA with Tukey's test with correction for multiple comparisons, (D) Two-way ANOVA with Šidák correction for multiple comparisons. (E, F, G, I) Two-way ANOVA with Tukey's test with correction for multiple comparisons. (H) Unpaired t test with Welch's correction. (I) Not all significant comparisons are shown. Asterisks indicate AdshCtrl TGF-β1 vs. AdshZNF697 TGF-β1 comparisons. ∗p<0.05, ∗∗p<0.01, ∗∗∗p<0.001, ∗∗∗∗p<0.0001. Error bars represent SD.

Inhibition of the TGF-β receptor I (ALK5) activity under basal conditions led to a strong reduction in *Zfp697* expression (Fig. 3C, Fig. S3D), suggesting that endogenous TGF-β receptor signaling contributes to the maintenance of basal *Zfp697* expression. TGF-β1 can trigger a canonical signaling pathway mediated by the Smad transcription factors, as well as multiple noncanonical pathways [64]. To investigate the contribution of the canonical TGF-β1 pathway to *Zfp697* expression, we treated hepatocytes with SIS3, an inhibitor of Smad3 phosphorylation and function [65]. Remarkably, this resulted in a complete abrogation of the induction of *Zfp697* expression by TGF-β1 in mouse primary hepatocytes (Fig. 3D), and in markedly lower expression levels in Huh7 cells (Fig. S3E). In line with this, siRNA-mediated knockdown of Smad4 nearly abolished TGF-β1-induced *Zfp697* expression and lowered basal levels, indicating that canonical TGF-β1/Smad signaling is necessary for both TGF-β1-responsiveness and maintenance of basal expression. Knockdown of Sox4, a transcriptional coregulator known to be induced by TGF-β1 and cooperate with, among others, Smad proteins [66], partially impaired the response (Fig. 3E, Fig. S3F). This suggests that Sox4 acts as a cooperating but non-essential downstream mediator of *Zfp697* regulation. In addition, published SMAD4 ChIP-seq data from HepG2 cells show SMAD4 binding at the *ZNF697* promoter region and intron 1 (Fig. S4A), conserved across multiple human and mouse cell types along with SMAD2, SMAD3, and SOX4 binding observed in ChIP-Atlas data [41], [42], [43] (Fig. S4B–C). Taken together, these results show that the canonical TGF-β1 signaling pathway is essential for *Zfp697* expression under both basal and stimulated conditions in mouse and human hepatocytes.

### 3.5 Zfp697 modulates the hepatic TGF-β1 response

Manipulation of Zfp697 expression affects the expression of genes encoding components of the TGF-β1 signaling pathway and TGF-β1 target genes in mouse primary hepatocytes (Fig. S2D and S2H), suggesting a role for Zfp697 in modulating the hepatic TGF-β1 response. Indeed, increasing Zfp697 expression in mouse primary hepatocytes significantly altered the transcriptional response to TGF-β1 treatment. Specifically, Zfp697 attenuated the TGF-β1-mediated upregulation of *Smad7*, *Cyp8b1*, *Tgfbr1*, *Fn1*, *Emp1*, and *Klf11* in addition to modulating the basal expression of the last four genes listed (Fig. 3F–G and Fig. S4D). To determine whether these transcriptional effects were accompanied by changes in canonical TGF-β1 signaling, we next examined TGF-β1-induced SMAD2 phosphorylation in Huh7 cells following manipulation of ZNF697 expression. Knockdown of ZNF697 significantly reduced acute TGF-β1-induced SMAD2 phosphorylation, whereas overexpression of ZNF697 had no detectable effect (Fig. 3H). These findings suggest that endogenous ZNF697 contributes to cellular responsiveness to TGF-β1, as reflected by SMAD2 phosphorylation, while increased ZNF697 expression alone is not sufficient to further enhance this response. Despite reduced p-SMAD2 levels, the effects of ZNF697 depletion on downstream gene expression are gene-specific, with some TGF-β1-responsive genes showing reduced induction (TGFBR1 and SMAD7) and others showing enhanced expression (ACTA2, CDKN1A, CYP8B1, and COL3A1) (Fig. 3I). These findings suggest that Zfp697 does not act as a simple positive regulator of TGF-β1 signaling but instead modulates the transcriptional and/or post-transcriptional response to TGF-β1 in a target-specific manner.

### 3.6 Hepatocyte-specific Zfp697 KO is associated with the absence of MASH-associated lean mass loss and improved glucose tolerance

To test the physiological role of hepatic Zfp697, we generated hepatocyte-specific Zfp697 knockout mice (HepKO) by crossing Zfp697 floxed mice [10] with mice expressing Cre driven by the albumin promoter [14]. We subjected HepKOs and floxed controls (Flox) to a 12-week diet- and chemical-induced MASH model involving a Western diet, fructose- and glucose-rich drinking water, combined with weekly CCl_4_ injections as described by Tsuchida and colleagues [15]. As illustrated in Fig. 4A, HepKO mice, along with Flox control littermates, were randomly assigned to the MASH model or the control group with chow diet and weekly injections of vehicle (corn oil). This generated four experimental groups: Flox Chow, HepKO Chow, Flox MASH, and HepKO MASH. We monitored body weights throughout the experiment and at the end assessed body composition, indirect calorimetry, glucose tolerance and collected plasma and tissues (Fig. 4A).

**Fig. 4:**
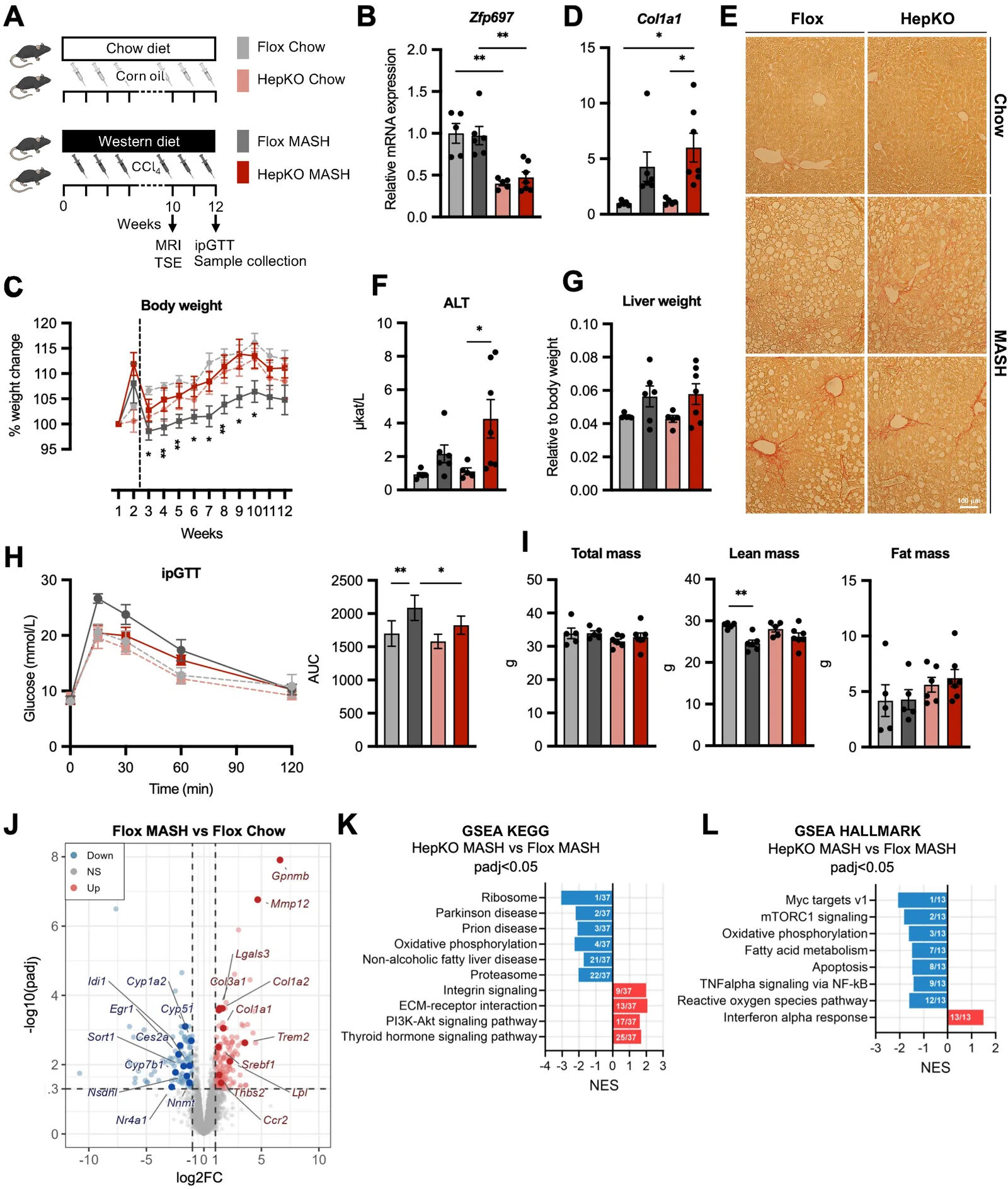
**Hepatocyte-specific Zfp697 knockout alleviates MASH-induced glucose intolerance.** (**A**) Experimental overview. (**B**) Zfp697 mRNA expression in liver (n = 5–7). (**C**) Body weight (n = 5–7). (**D**) Col1a1 mRNA expression in liver (n = 5–7) (**E**) Representative liver sections stained with picrosirius red. (**F**) Plasma ALT levels (n = 5–7). (**G**) Liver weight relative to body weight (n = 5–7). (**H**) Glucose tolerance test (n = 5–7). (**I**) Total, lean, and fat mass (n = 5–7). (**J**) Differential expressed genes in Flox MASH versus Flox Chow liver tissue. (**K**) Gene Set Enrichment Analysis (GSEA) of KEGG pathways and (**L**) Hallmark gene sets comparing HepKO MASH and Flox MASH liver samples. Normalized enrichment scores (NES) are shown for significantly enriched pathways. Statistics: (B, D) Two-way ANOVA with Tukey's test with correction for multiple comparisons, (C, F–I) One-way ANOVA with Tukey's test with correction for multiple comparisons. ∗p<0.05, ∗∗p<0.001, ∗∗∗p<0.0001. Error bars represent SEM.

First, we confirmed the reduction in liver *Zfp697* expression in HepKOs by approximately 60% (Fig. 4B). The remaining 40% is likely derived from non-hepatocyte populations, consistent with publicly available single-cell RNA-seq data (GEO: GSE192742) [45], which show *Zfp697* expression not only in hepatocytes but also in e.g. endothelial cells, cholangiocytes, and HSCs/progenitor cells (HsPCs) (Fig. S5A). Next, we examined the body weights, which across groups tended to increase throughout the experiment, reflecting the combined obesogenic effect of the Western diet and body weight lowering effect of CCl_4_ (clearly noticeable after the first injection, dashed line) [15], [67], [68] (Fig. 4C). As expected [15], [67], [68], body weights of Flox MASH mice remained significantly lower throughout weeks 3–10 when compared to chow-fed controls. However, the body weight of HepKO MASH mice remained similar to chow-fed controls, indicating that the loss of hepatocyte Zfp697 expression provides a protective effect against the liver injury- and catabolic stress-induced weight loss characteristic of this MASH diet [15].

In line with previous observations, the experimental MASH model displayed molecular and histological features consistent with fibrotic remodeling, including increased picrosirius red staining and elevated hepatic expression of the fibrosis-associated marker *Col1a1* (Fig. 4D-E). MASH mice also exhibited elevated, or trending elevated, plasma levels of the liver enzymes ALT, AST, and GLDH (Fig. 4F, S5B), and a nominal (albeit not significant) increase in liver weight (Fig. 4G, Fig. S5C–D). We did not observe significant differences between genotypes for any of these particular parameters, indicating they did not differentiate between the degree of injury and fibrosis in HepKO and Flox mice. Nonetheless, and in line with the protection from diet-induced weight loss, HepKO MASH mice did not develop glucose intolerance when compared to controls, as opposed to the Flox MASH (Fig. 4H). In addition, body composition analysis at week 10 showed that Flox MASH mice had significantly lower lean mass compared to chow-fed Flox controls, which was not observed in HepKO mice (Fig. 4I). Given that liver weights were similar between MASH groups (Fig. 4G), the absence of a MASH-associated reduction in lean mass in HepKO MASH mice indicates that tissues other than the liver contributed to this phenotype.

To assess whether differences in energy metabolism contributed to the observed phenotypes, we performed indirect calorimetry to examine potential differences in energy expenditure, substrate preference, and locomotor activity across genotypes and diets. After acclimation, mice were monitored for 48 h for O_2_ consumption, CO_2_ production, energy expenditure, respiratory exchange ratio, food intake, drink intake, energy balance, and locomotor activity (Fig. S5E-O). ANCOVA tests using lean mass, fat mass, and total mass as covariates, as well as ANOVA tests, found no statistically significant differences elicited by genotype or diet. As expected, there was a trend suggesting that MASH mice rely more on lipid oxidation as fuel source, as reflected by the lower RER during dark phases (Fig. S5F–G), and have a higher total calorie intake and energy balance (Fig. S5L-M), consistent with the differences in diet composition. Thus, the absence of a significant reduction in lean mass and the improved glucose tolerance observed in HepKO MASH mice are unlikely to be explained by differences in systemic energy balance or substrate utilization.

RNA-seq analysis of liver tissue confirmed the expected MASH-associated transcriptional signature, characterized by increased expression of genes associated with macrophage activation and fibrosis, including *Gpnmb*, *Mmp12*, *Col1a1*, *Col1a2*, *Trem2*, and *Lgals3*, together with reduced expression of genes associated with normal hepatocyte metabolic function, including *Cyp7b1*, *Cyp51*, *Cyp1a2*, *Idi1*, and *Nr4a1* (Fig. 4J, Table S2). Over-representation analysis revealed that the transcriptional response to MASH was dominated by pathways involved in lipid and cholesterol metabolism as well as fibrosis-associated ECM remodeling (Fig. S6A), consistent with enrichment of liver disease-related gene signatures (Fig. S6B). Similar transcriptional changes were observed in HepKO mice exposed to the MASH diet (Fig. S6C–D), indicating that hepatocyte-specific deletion of Zfp697 does not substantially alter the overall hepatic response to MASH at the individual gene level.

To directly investigate genotype-dependent effects during MASH, we compared HepKO and Flox mice under MASH conditions. Gene Set Enrichment Analysis (GSEA) identified coordinated changes in multiple biological pathways. Relative to Flox MASH mice, HepKO MASH mice exhibited enrichment of pathways related to ECM organization, integrin signaling, PI3K-AKT signaling, thyroid hormone signaling, and interferon-α response, while pathways associated with oxidative phosphorylation, fatty acid metabolism, mTORC1 signaling, ribosome function, proteasome activity, TNFα–NF–κB signaling, and reactive oxygen species were reduced (Fig. 4K-L, Table S2).

Taken together, in experimental MASH, hepatocyte-specific ablation of Zfp697 is associated with the absence of MASH-associated lean mass loss, improved glucose tolerance, and stable body weight. However, its effects on hepatic outcomes are mixed, with no alleviation of hepatic fibrosis or prevention of the canonical MASH transcriptional program. Transcriptomic analyses revealed selective remodeling of inflammatory pathways, characterized by enrichment of an interferon-α response signature and reduced TNFα–NF–κB and oxidative stress-associated signatures.

### 3.7 Zfp697 interacts with protein complexes involved in RNA processing and translation

To gain further insight into the function of Zfp697 we sought to identify its protein binding partners in hepatocytes. We started by expressing and purifying Zfp697 with an N-terminal glutathione S transferase (GST) fusion. We then used GST-Zfp697 or GST alone (background control) as bait to affinity purify interacting proteins from a mouse hepatocyte nuclear-enriched protein extract, followed by protein identification via liquid chromatography-tandem mass spectrometry (LC-MS/MS) (Fig. 5A). Quality control of replicate samples revealed one outlier (Fig. S7A), which was removed from subsequent analysis (Fig. 5B). We filtered the data to include proteins that appeared in at least two GST-Zfp697 replicates and either had a log_2_ fold change (FC) > 1 and adjusted p-value (p.adj) < 0.05 or were uniquely detected in the GST-Zfp697 samples. With these criteria we identified 425 proteins enriched in the GST-Zfp697 samples constituting Zfp697 interactors (Table S3). Gene ontology (GO) analysis revealed significant enrichment of proteins involved in translation, mRNA processing, RNA splicing, and ribosomal subunit assembly (Fig. 5C, first panel). Enriched cellular components included intracellular ribonucleoprotein complexes and ribosomes (second panel), while molecular functions highlighted RNA binding and ribosomal structural proteins (third panel). Additionally, protein domains related to nucleotide binding and RNA recognition motifs (RRM) were significantly enriched (last panel). Interestingly, and in line with their role in mRNA processing, splicing, and translation, most Zfp697 interactors shuttle between nucleus and cytoplasm (Fig. S7B).

**Fig. 5:**
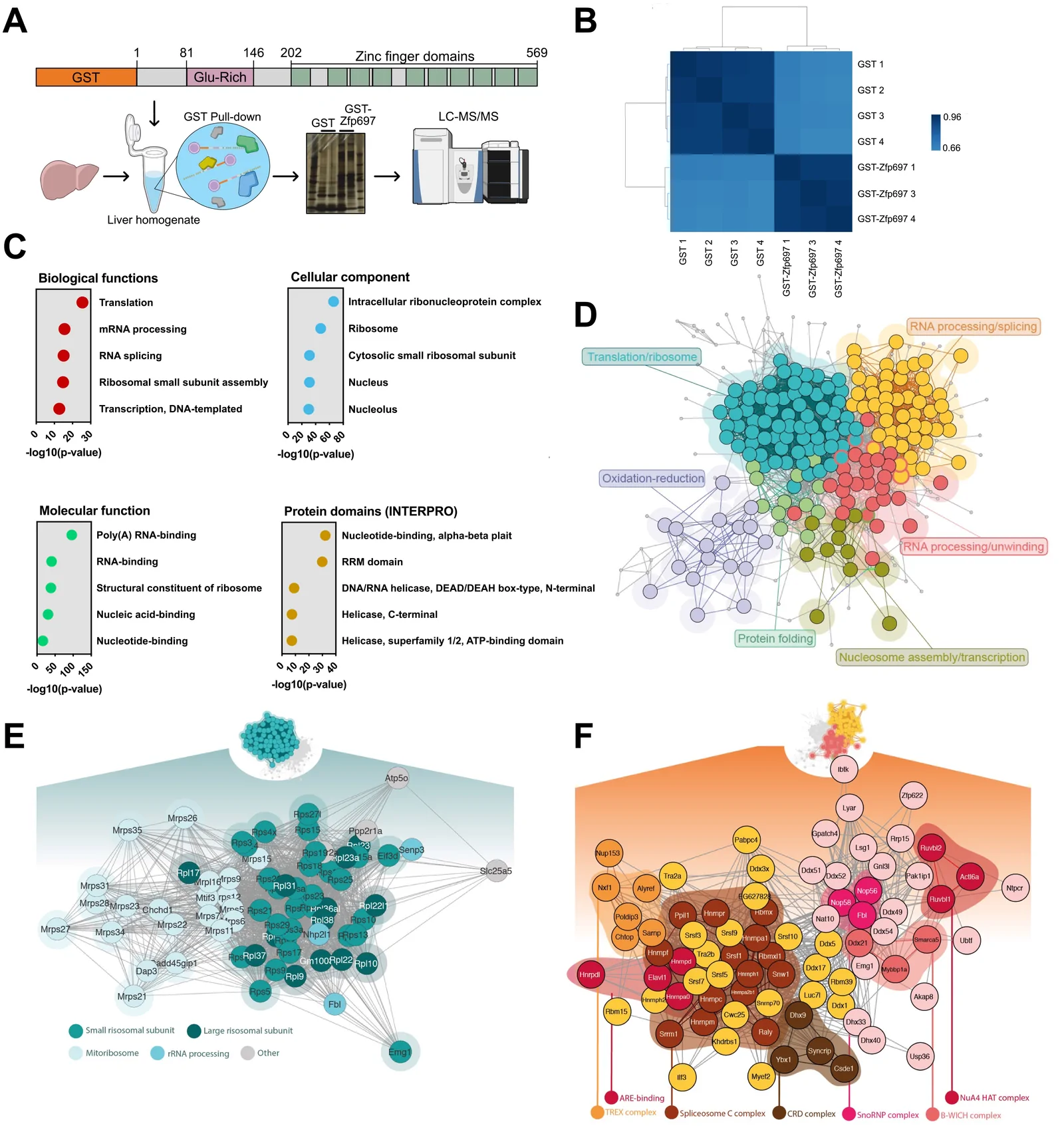
**Zfp697 interacts with RNA-processing complexes.** (**A**) Experimental overview. (**B**) Clustermap of pairwise Pearson correlations between 4 GST and 3 GST-Zfp697 samples. (**C**) DAVID Gene Ontology (GO) term analysis of GST-Zfp697 interactors, showing enriched biological processes, cellular components, molecular functions, and domains. (**D**) STRING network of Zfp697 interactors clustered into six functional groups by ClusterOne. (**E-F**) Detailed networks of (**E**) translation/ribosome cluster and (**F**) RNA processing/splicing/unwinding clusters. Grey lines represent curated interactions, circle colors indicate protein families, and background shading denotes protein complexes. Distances between proteins reflect interaction strength and confidence.

To get further insight into molecular function(s) we performed network analysis on the Zfp697 interactome. First, we established connections between these proteins using the STRING protein–protein interaction database [31] and built a protein–protein interaction network based on these data using Cytoscape [33]. We then performed unbiased clustering using ClusterOne, which identified six main clusters of Zfp697 interactors that interact amongst themselves (Fig. 5D). GO analysis revealed that the three major clusters identified are associated with RNA translation, splicing, and unwinding, with three smaller clusters comprising proteins related to oxidation-reduction, protein folding and nucleosome assembly and transcription. As examples, in the translation and ribosome cluster, Zfp697 interacted with several proteins of the Rps, Rpl, and Mrps families, which constitute structural components of the small and large ribosomal subunit and mitochondrial ribosomes (Fig. 5E). The two other major clusters: RNA processing, splicing and unwinding, include many RNA-binding proteins, several of which belong to critical RNA-processing complexes (Fig. 5F). These includes the TRanscription-EXport (TREX) complex, which couples mRNA transcription with nuclear export [69], [70], the coding region determinant (CRD)-mediated complex, which binds and stabilizes mRNA molecules [71], and the spliceosome C complex, which catalyzes the second transesterification step in pre-mRNA splicing, resulting in intron removal and exon ligation [72].

This pull-down-proteomics approach defined the hepatocyte Zfp697 interactome and revealed interactions with complexes that govern multiple aspects of RNA biology, across RNA life cycle, including transcription, splicing, transport, stability, and translation.

### 3.8 Liver Zfp697 interacts with multiple RNAs

Since Zfp697 binds directly to RNA [10] and interacts with multiple RNA-interacting complexes, we sought to investigate Zfp697's direct interaction with RNAs in the liver using enhanced crosslinking and immunoprecipitation (eCLIP), followed by sequencing. As no antibodies are currently available that specifically recognize Zfp697, we expressed FLAG-tagged Zfp697 via adenovirus delivery to the livers of C57BL/6J male mice. As illustrated in Fig. 6A, we performed eCLIP with FLAG immunoprecipitation (IP) on the liver tissue and analyzed data using Skipper [21]. We first confirmed the higher exogenous *Zfp697* expression by RT-qPCR, while endogenous levels remained unaffected, as measured by primers targeting the Zfp697 exon 2-3 junction and Zfp697 3′UTR region, respectively (Fig. 6B). Moving on to the eCLIP-seq analysis, Skipper tiles >100 nucleotide windows across the transcriptome and test for enrichment in IP samples over input. Skipper-generated quality control analysis showed that the total number of enriched and non-enriched windows in the eCLIP was reproducible among the three replicates (Fig. 6C). Using the filtering: pmax <0.05 and mean log_2_ fold enrichment >3, we identified 1,213 significantly reproducible enriched windows (Fig. 6D, Table S4). These windows represent over 1,000 distinct RNAs, the majority of which are protein coding transcripts (74.6%), but also include other transcript types such as miRNAs, including miR-21 and miR-122 (Fig. S8A, Table S4). Most enriched windows cover RNA introns (42.5%), while others span exons of non-coding RNAs or splicing sites (Fig. 6E). For example, we found that Zfp697 interacts with the introns of *Cdk6* (Fig. 6F), *Cxcr2*, *Cdk19*, *Col14a1*, the coding sequence of *Il1r2*, and the near the 5′ splice site of *Il17d* (Fig. S8B). As exemplified by these targets, Zfp697 interaction occurs in narrow peaks of ∼25 bp, and motif analysis of all fine-mapped windows identified three significantly enriched motifs (Fig. 6G). In addition, we find that Zfp697 binds several RNAs associated with the modulation of TGF-β1 signaling. This includes *Ltbp2*, which influences TGF-β1 availability [73]; *Furin*, which processes latent TGF-β1 into its active form [74], [75], *Usp15*, a deubiquitylase that stabilizes TGF-β receptor I [76]; and *Nedd4l*, which regulates TGF-β receptor turnover through ubiquitination [77] (Table S4). Although Zfp697 interacts with these injury-response RNAs, and TGF-β1 pathway modulators, transcriptional regulation emerged as the dominant GO category among the Zfp697-bound RNAs (Fig. S8C).

**Fig 6:**
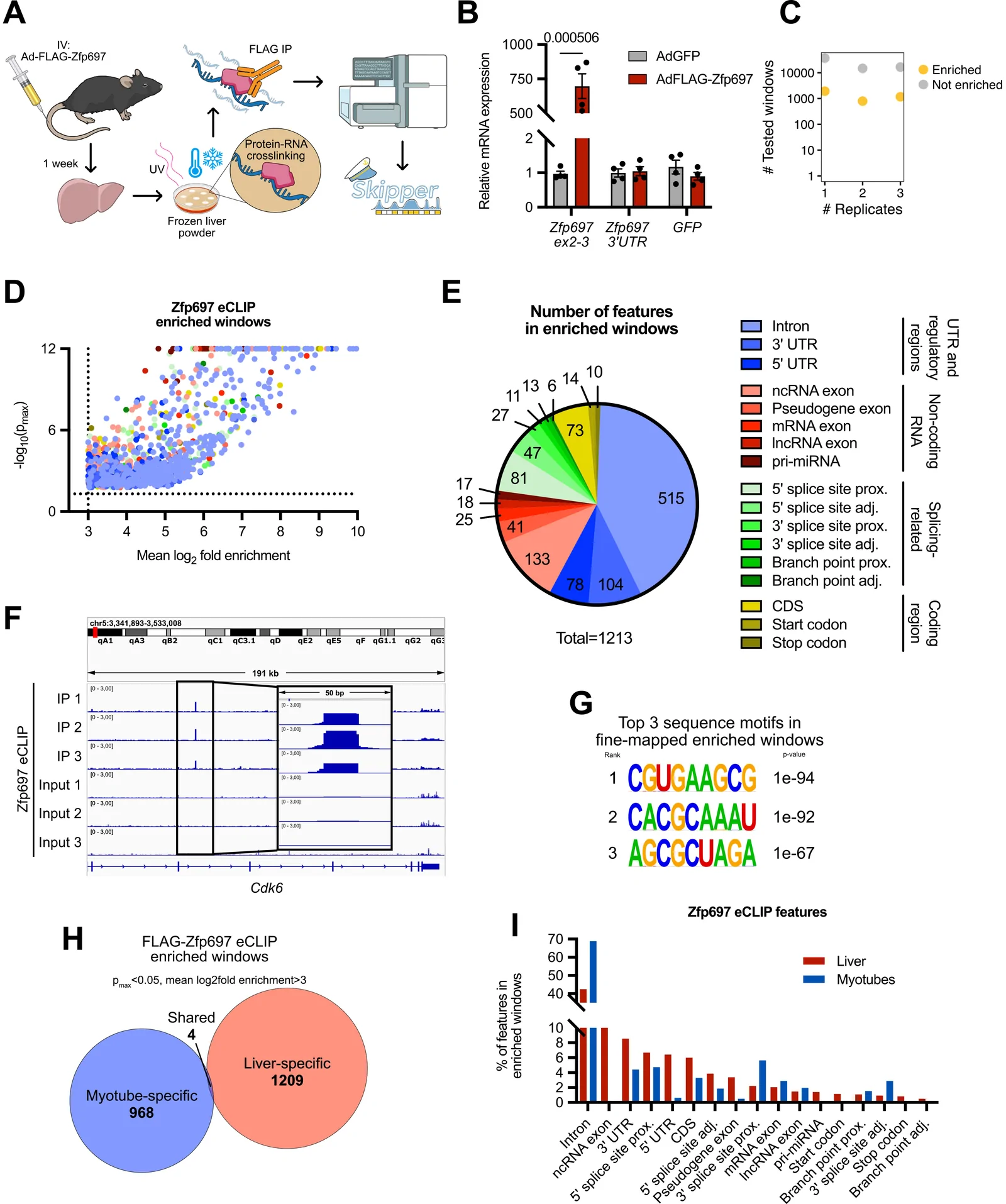
**Zfp697 interacts with multiple RNAs in mouse liver.** (**A**) Experimental overview. (**B**) mRNA expression of translated (exon 2–3), untranslated (3′UTR) regions of *Zfp697* and *GFP* in livers of AdFLAG-Zfp697 and AdGFP injected mice (n = 4). (**C**) Skipper QC showing reproducible enriched windows among the three eCLIP replicates. (**D**) Distribution of the 1,213 enriched windows (p < 0.05 and log_2_ fold enrichment >3). (**E**) Classification of features in enriched windows. (**F**) IGV browser view of Zfp697 interaction with *Cdk6* intronic region. (**G**) Top 3 motifs from HOMER *de novo* motif analysis of fine-mapped enriched windows. (**H–I**) Comparison of FLAG-Zfp697 eCLIP-seq data in liver (this study) with previously published eCLIP data from primary mouse myotubes: (**H**) Overlap of enriched windows and (**I**) feature distribution within enriched windows. Statistics: (B) Student's unpaired t-tests with FDR correction, q values < 0.05 shown. Error bars represent SEM.

Given that Zfp697 also interacts with RNAs in myotubes [10], we imported and reanalyzed those raw files using the same pipeline to investigate the cell or tissue-specificity of Zfp697's RNA-interacting function. Applying the same filtering criteria, we find 972 enriched windows in primary mouse myotubes, of which only four were shared with liver: in the RNAs of *Fsd1l*, *Foxe1*, *Polr1e*, and *n-TSaga9* (Fig. 6H). When comparing the features in the enriched windows, we observed an even stronger preference for intronic regions in the myotubes (69%) compared to the liver (42.5%), while “ncRNA exon” and “5’UTR” features were absent or nearly absent in the myotubes (Fig. 6I). In addition, motif analysis of the enriched windows in myotubes revealed no similarity to the motifs found in liver (Fig. S8D).

These results show that Zfp697 interacts with multiple context-dependent RNAs, predominantly protein-coding transcripts, and primarily within intronic regions.

Taken together, our proteomic and eCLIP analysis support a role for Zfp697 in RNA metabolism and suggest it may function as an RNA-interacting regulator in the hepatic injury and disease response.

## 4 Discussion

Identifying key drivers and regulators of hepatic disease and inflammatory responses can help uncover underlying mechanisms, identify biomarkers, and reveal potential therapeutic targets. In this study, we find that the mRNA levels of *ZNF697/Zfp697* are associated with liver disease and injury, correlating with multiple known markers of inflammation. Among the large family of ZFPs, a few other have previously been implicated or linked to liver diseases such as steatosis, MASH, and hepatocellular carcinoma, including ZBTB20/Zbtb20 [78], [79], ZNF638/Znf638 [80], ZFP281/ZNF281 [81], and ZEB1 [16], [17]. Interestingly, we identify Zbtb20 and Znf638, both regulators of hepatic lipogenesis, as Zfp697 protein interactors (Fig. S7B). These two proteins rank among the top 100 Zfp697 interactors (ranked by significance) (Table S3), which also includes the liver isoform of Carnitine O-palmitoyltransferase 1 (Cpt1a). CPT1A plays a pivotal role in lipid metabolism, primarily by facilitating the transport of long-chain fatty acids into the mitochondria for beta-oxidation [82]. These data suggest that Zfp697 may have additional roles in the control of hepatic energy metabolism, possibly mediated through protein–protein interactions.

*Zfp697* expression levels show a strong positive correlation with transcripts related to inflammation, stress response, and ECM remodeling. In line with this, we found that *Zfp697/ZNF697* expression is controlled by the canonical TGF-β1 signaling pathway and that Zfp697 influences TGF-β1 pathway responses at both the signaling level, as reflected by changes in SMAD2 phosphorylation, and at the transcriptional level through modulation of TGF-β1-responsive genes. TGF-β is a major mediator of liver inflammation, ECM remodeling, and fibrosis, involved in the progression from acute to chronic liver injury. This includes the well-established role of TGF-β in activating HSCs, a central event in liver fibrosis [64]. However, TGF-β signaling in hepatocytes has also been shown to contribute to hepatocyte death and lipid accumulation, and its selective inhibition in hepatocytes can attenuate hepatic fibrosis [83], [84].

In this regard, modulation of TGF-β signaling has been suggested as a potential therapeutic target for treating acute liver injury. For example, inhibition of TGF-β1 receptor 1 has been shown to enhance liver regeneration and improve mouse survival in models of liver injury and senescence [36] and several clinical trials of TGF-β-targeting therapies are currently ongoing [64]. We find that increasing Zfp697 expression in mouse primary hepatocytes reduces the expression of genes encoding components of the TGF-β signaling pathway (Fig. S2D), as well as the effects of TGF-β1 on the expression of classical target genes (Fig. 3F-G). Conversely, inhibition of ZNF697 expression increased TGF-β1 response on these, while expression of genes encoding TGF-β signaling components was downregulated (Fig. 3I) indicating that ZNF697 influences TGF-β1-responsive transcriptional programs. The reduced TGF-β1-induced SMAD2 phosphorylation following ZNF697 depletion further supports a role for ZNF697 in modulating cellular responsiveness to TGF-β1; however, these findings do not establish effects on TGF-β1 production, secretion, or activation. Although our data identify clear links between Zfp697 expression and TGF-β1 signaling, the relationship is more complex than a simple linear association. Rather than supporting a direct relationship between Zfp697 expression and TGF-β-associated gene expression, our findings suggest that Zfp697 modulates these in a context-dependent manner. Our findings raise the interesting possibility that Zfp697 regulates TGF-β1 signaling at multiple levels. Future studies will be important to further define the molecular mechanisms underlying this regulation and determine the relative contributions of ligand activity, receptor signaling, SMAD phosphorylation, post-transcriptional regulation, and other regulatory processes within the pathway. Given Zfp697's role in modulating TGF-β1 signaling, targeting Zfp697 may offer a novel strategy to influence inflammation, fibrosis, or aberrant immune responses, which are hallmarks of liver and other chronic diseases.

Our data demonstrates that Zfp697 functions as a bona fide RNA-interacting protein in the liver, supporting previous observations in myotubes [10]. This aligns with recent large-scale analyses identifying widespread RNA-binding activity among ZFPs, particularly those harboring C2H2 domains [8], [9]. Despite Zfp697 having a similar function in myotubes; modulating injury, inflammation, and tissue repair response [10], it interacts with distinct sets of RNAs in each tissue (Fig. 6H), albeit with largely overlapping feature preferences (Fig. 6I). Moreover, the enriched RNA sequence motifs identified in liver (Fig. 6G) and myotubes (Fig. S8D) differ, suggesting that Zfp697 may exhibit sequence-specific RNA interaction but in a tunable, context-sensitive way, or perhaps rather associates with RNAs either via interactions with tissue-specific RBPs or by recognizing secondary RNA structures.

We find that Zfp697 predominantly interacts with intronic regions (Fig. 6I), indicating a role in co-transcriptional or early RNA processing events in the nucleus. Consistent with this, many of its protein interaction partners are nucleocytoplasmic RNA-binding proteins (Fig. S7B), implicating Zfp697 in dynamic ribonucleoprotein complexes that assemble in the nucleus and are maintained or remodeled in the cytoplasm. This suggests that Zfp697 may shuttle between compartments and function in both nuclear RNA processing and post–export regulation.

Interestingly, although only a subset of Zfp697-bound transcripts encodes canonical components or regulators of the TGF-β pathway, many additional targets are involved in RNA processing, receptor trafficking, cytoskeletal organization, extracellular matrix remodeling, and cellular signaling. These findings suggest that Zfp697 may coordinate broader post-transcriptional programs that shape hepatocyte responses to TGF-β1 and other stress-associated signaling pathways, rather than acting exclusively on a single signaling cascade. Together, these findings are consistent with the possibility that Zfp697 influences TGF-β signaling at multiple levels, although additional studies will be required to determine which of these direct RNA interactions are functionally relevant. The RNA-binding and proteomic datasets suggest that Zfp697 is unlikely to solely function as a dedicated regulator of the canonical TGF-β pathway. Instead, Zfp697 appears to associate with RNA-processing complexes and bind transcripts encoding multiple signaling and transcriptional regulators, including components linked to TGF-β, PDGF, BMP, FGF, and extracellular matrix remodeling. This broader interaction landscape is consistent with Zfp697 acting as a post-transcriptional integrator of hepatocyte stress responses, with modulation of TGF-β signaling representing one manifestation of this wider regulatory function.

This work paves the way for future investigations into the relationship between Zfp697's RNA-interacting activity and its regulation of TGF-β1 signaling and liver disease and injury response. Notably, among the miRNAs identified as Zfp697 interactors in our eCLIP data, several, such miR-21 and miR-122, are known regulators of the TGF-β1 signaling and of liver inflammation and fibrosis [49], [85], [86], [28].

Despite its aberrant expression in liver injury and disease and correlation with inflammatory and fibrotic gene signatures, hepatocyte-specific Zfp697 ablation did not uniformly improve hepatic outcomes, with no improvement in liver fibrosis or biomarkers of liver damage. However, Zfp697 HepKO mice exhibited differences in their response to experimental MASH, characterized by the absence of MASH-associated lean mass loss, stable body weight, and improved glucose tolerance. Furthermore, RNA-seq analysis suggests that the hepatocyte-specific loss of Zfp697 alters hepatic metabolic programs including PI3K-Akt, AMPK, mTORC1 signaling, oxidative phosphorylation, and fatty acid metabolism, that may contribute to the improved glucose tolerance and provide hypotheses for future mechanistic studies. Although our *in vitro* studies demonstrate that Zfp697 influences TGF-β1 signaling, hepatocyte-specific deletion of Zfp697 *in vivo* did not produce a dominant TGF-β pathway transcriptional signature in the MASH model. Instead, RNA-seq identified changes in metabolic, bioenergetic, extracellular matrix, and inflammatory signaling pathways. Whether these changes reflect alterations in individual cytokine levels or activity, including IFN-γ, IL-6, and active TGF-β1, remains to be determined. Thus, the broader transcriptional response observed *in vivo* suggests that the consequences of Zfp697 deficiency extend beyond the modulation of TGF-β1 signaling alone and likely reflect the integration of multiple stress-response programs during chronic liver injury. Several factors may explain the differences between the *in vitro* and *in vivo* findings. Whereas the *in vitro* experiments examined the acute response of isolated hepatocytes to TGF-β1, the MASH model reflects a chronic, multifactorial disease in which compensatory mechanisms and crosstalk with non-parenchymal liver cells may buffer the effects of hepatocyte-specific Zfp697 deletion. Moreover, RNA-seq captures a single disease stage and may therefore miss transient changes in TGF-β pathway activity.

Interestingly, while we observed robust increase in *Zfp697* expression in the MCD, FF, and CCl_4_ models among others (Fig. 1), *Zfp697* was not elevated in the MASH model at time of termination (Fig. 4B). This may reflect differences in the temporal dynamics of Zfp697 regulation, as injury-responsive genes can show transient induction during early disease stages followed by adaptation or normalization during chronic disease progression. Importantly, the functional effects observed following hepatocyte-specific Zfp697 deletion indicate that Zfp697 contributes to disease-associated processes in MASH despite the absence of sustained expression elevation at the late disease stage.

While a Western diet promotes substantial weight gain, the hepatotoxin CCl_4_ induces a strong systemic inflammatory response that can drive muscle wasting and weight loss, even in the context of calorically dense diets [15], [87], [88]. Given Zfp697's role in inflammatory signaling, its hepatocyte-specific deletion may reduce liver-derived pro-inflammatory or catabolic signals, helping to preserve skeletal muscle mass and support systemic insulin sensitivity. Although the specific lean tissue(s) responsible for the observed differences in lean mass could not be determined because individual tissue weights were not measured, skeletal muscle - which constitutes the majority of lean mass in mice - is likely to be a major contributor. The preserved glucose tolerance further supports this possibility. The lack of significant differences in calorie intake, substrate utilization, and energy expenditure (Fig. S5E-O), suggests that the absence of a reduction in lean mass and the improved glucose tolerance observed in HepKO mice are unlikely due to altered energy balance but rather to hepatocyte-specific, Zfp697-dependent mechanisms. Thus, our findings support a role of Zfp697 in orchestrating hepatic stress responses and inflammatory cues that influence whole-body metabolism. Interestingly, the effects of hepatocyte-specific Zfp697 deletion on body weight progression (Fig. 4C) became less pronounced during the final two weeks of the study. This attenuation suggests that compensatory mechanisms or adaptation to chronic disease may reduce the impact of Zfp697 deficiency over time. As most endpoint analyses were performed after this period, future studies incorporating additional intermediate time points may help define the temporal contribution of Zfp697 to MASH progression.

Our HepKO model reduces *Zfp697* expression by ∼60% in the liver (Fig. 4B), as deletion is restricted to hepatocytes. However, other liver cell populations also express Zfp697 (Fig. S5A), raising the possibility that Zfp697 may have important functions outside the hepatocyte compartment. For example, activated HSCs are central to liver fibrosis progression [54], and we find that *ZNF697/Zfp697* expression is increased in HSCs upon injury, fatty acid accumulation, and TGF-β1 signaling (Fig. S1K-M). Thus, the absence of a strong anti-fibrotic phenotype following hepatocyte-specific Zfp697 deletion does not exclude a role for Zfp697 in fibrosis-related processes mediated by other liver cell populations. As the present model selectively deletes Zfp697 in hepatocytes while preserving its expression in non-parenchymal liver cells and extrahepatic tissues, our findings define the contribution of hepatocyte Zfp697 but do not address potential functions in other cellular compartments. Future studies using HSC-specific or combined cell-type-specific deletion models will be important to determine the contribution of Zfp697 to ECM remodeling, fibrosis, and liver disease progression.

For the first time, we characterize ZNF697/Zfp697 in liver and hepatocytes investigating it's *in vitro* and *in vivo* contributions to liver disease adaptations. We find that high *ZNF697/Zfp697* expression can be used as a marker for a range of chronic and acute liver diseases and injuries. We show that ZNF697/Zfp697 binds RNA in a context-dependent manner, interacts with extensive RNA-processing machinery, and modulates the expression of genes involved in inflammation, ECM remodeling, fibrosis, and metabolism. We further demonstrate that ZNF697/Zfp697 is induced by canonical TGF-β1 signaling and modulates the hepatic transcriptional response to TGF-β1. Interestingly, the effects of hepatocyte-specific Zfp697 ablation in experimental MASH are mostly reflected at the systemic level, with heterogeneous effects on hepatic outcomes. The mechanisms underlying these systemic effects may involve yet uncharacterized cell and tissue crosstalk.

## CRediT authorship contribution statement

**Stine M. Præstholm:** Writing – original draft, Visualization, Validation, Methodology, Investigation, Funding acquisition, Formal analysis, Data curation, Conceptualization. **Jorge C. Correia:** Writing – review & editing, Visualization, Validation, Methodology, Investigation, Funding acquisition, Formal analysis, Data curation, Conceptualization. **Rafael Hoyos Justiniano:** Investigation. **Alisson L. da Rocha:** Investigation, Formal analysis. **Aurel B. Leuchtmann:** Validation, Investigation. **Tadas Sereiva:** Investigation. **Margareta Porsmyr-Palmertz:** Validation, Investigation. **Kathrine Rothamel:** Investigation, Formal analysis, Data curation. **Dorota Kaminska:** Investigation, Formal analysis, Data curation. **Ranjani Sri Ganji:** Investigation, Formal analysis, Data curation. **David Potěšil:** Visualization, Investigation, Formal analysis, Data curation. **Zbyněk Zdráhal:** Writing – review & editing, Supervision, Resources, Project administration. **Jussi Pihlajamäki:** Writing – review & editing, Supervision, Resources, Project administration. **Gene W. Yeo:** Writing – review & editing, Supervision, Resources, Project administration. **Jorge L. Ruas:** Writing – original draft, Supervision, Funding acquisition, Conceptualization.

## Ethics declaration

This study was conducted in accordance with the ARRIVE (Animal Research: Reporting of In Vivo Experiments) guidelines. This study was approved by the regional animal ethics committee of Northern Stockholm, Sweden. (Approval No. 4039–2018 & 4359–2020).

## Data availability

The 3’-RNA-seq data is deposited at GEO: GSE329899 (https://www.ncbi.nlm.nih.gov/geo/query/acc.cgi?acc=GSE329899).

The mass spectrometry proteomics data have been deposited in the ProteomeXchange Consortium via the PRIDE [90] partner repository with the dataset identifier PXD064734 (https://www.ebi.ac.uk/pride/archive/projects/PXD064734). eCLIP is deposited at GEO: GSE302600 (https://www.ncbi.nlm.nih.gov/geo/query/acc.cgi?acc=GSE302600). All other data are available upon request. Unique reagents generated in this study will be made available upon request to the Lead Contact with a completed Materials Transfer Agreement.

## Funding

This work was supported by grants from The Swedish Research (2016–00785 and 2022–02743 to J.L.R), the 10.13039/501100009708Novo Nordisk Foundation (NNF21OC0070149 and NNF19OC0054132 to J.L.R), and the 10.13039/501100009782Ruth and Richard Julin Foundation (2022-00269 and 2023-00315 to S.M.P), The 10.13039/501100007743European Society for Clinical Nutrition and Metabolism (Research Fellowship to J.C.C) and 10.13039/501100009782Ruth and Richard Julin Foundation (to J.C.C). S.M.P. received a postdoctoral fellowship from the Strategic Research Programme (10.13039/100024950SRP) in Diabetes at Karolinska Institutet. T.S. was supported by 10.13039/100000002NIH T32-NS076401 and A.B.L. received the Frankel IHBH Inaugural Postdoctoral Fellowship Award 2026. CIISB, Instruct-CZ Centre of Instruct-ERIC EU consortium, funded by MEYS CR infrastructure project LM2023042 and European Regional Development Fund-Project “Innovation of Czech 10.13039/100031425Infrastructure for Integrative Structural Biology” (No. CZ.02.01.01/00/23_015/0008175), is gratefully acknowledged for the financial support of the measurements at the 10.13039/501100006435CEITEC Proteomics Core Facility. Computational resources were provided by the e-10.13039/100010666INFRA CZ project (ID: 90254), supported by MEYS CR.

## Declaration of competing interest

The authors declare the following financial interests/personal relationships which may be considered as potential competing interests: Jorge L. Ruas reports financial support was provided by Swedish Research Council. Jorge L. Ruas reports financial support was provided by Novo Nordisk Foundation. Stine M. Praestholm and Jorge C. Correia reports financial support was provided by Ruth and Richard Julin Foundation. Jorge C. Correia reports financial support was provided by European Society for Clinical Nutrition and Metabolism. Tadas Sereiva reports financial support was provided by National Institutes of Health. Aurel B. Leuchtmann reports financial support was provided by Frankel IHBH. Ranjani Sri Ganji David Potesil Zbynek Zdrahal reports financial support was provided by European Regional Development Fund. Gene W. Yeo reports a relationship with Jumpcode Genomics, Inc. That includes: board membership. Gene W. Yeo reports a relationship with Eclipse Bioinnovations Inc that includes: board membership, consulting or advisory, and equity or stocks. Stine M. Praestholm reports a relationship with Gubra AS that includes: employment. Jorge C. Correia reports a relationship with Novo Nordisk Research Centre Oxford that includes: employment. If there are other authors, they declare that they have no known competing financial interests or personal relationships that could have appeared to influence the work reported in this paper.

## References

[1] Hora S., Wuestefeld T. (2023). Liver injury and regeneration: current understanding, new approaches, and future perspectives. Cells.

[2] Campana L., Esser H., Huch M., Forbes S. (2021). Liver regeneration and inflammation: from fundamental science to clinical applications. Nat Rev Mol Cell Biol.

[3] Sharma S., Le Guillou D., Chen J.Y. (2023). Cellular stress in the pathogenesis of nonalcoholic steatohepatitis and liver fibrosis. Nat Rev Gastroenterol Hepatol.

[4] Younossi Z.M., Kalligeros M., Henry L. (2025). Epidemiology of metabolic dysfunction-associated steatotic liver disease. Clin Mol Hepatol.

[5] Cassandri M., Smirnov A., Novelli F., Pitolli C., Agostini M., Malewicz M. (2017). Zinc-finger proteins in health and disease. Cell Death Discov.

[6] Peng Q., Li J., Wu Q., Wang P., Kang Z., Deng Y. (2023). ZNF385A and ZNF346 serve as prognostic biomarkers associated with an inflamed immunosuppressive tumor microenvironment in hepatocellular carcinoma. Int J Mol Sci.

[7] Cook M.E., Bradstreet T.R., Webber A.M., Kim J., Santeford A., Harris K.M. (2022). The ZFP36 family of RNA binding proteins regulates homeostatic and autoreactive T cell responses. Sci Immunol.

[8] Gosztyla M.L., Zhan L., Olson S., Wei X., Naritomi J., Nguyen G. (2024). Integrated multi-omics analysis of zinc-finger proteins uncovers roles in RNA regulation. Mol Cell.

[9] Nabeel-Shah S., Pu S., Burns J.D., Braunschweig U., Ahmed N., Burke G.L. (2024). C2H2-zinc-finger transcription factors bind RNA and function in diverse post-transcriptional regulatory processes. Mol Cell.

[10] Correia J.C., Jannig P.R., Gosztyla M.L., Cervenka I., Ducommun S., Præstholm S.M. (2024). Zfp697 is an RNA-binding protein that regulates skeletal muscle inflammation and remodeling. Proc Natl Acad Sci U S A.

[11] Guo X., Deng Y., Jiang W., Li H., Luo Y., Zhang H. (2025). Single cell transcriptomic analysis reveals tumor immune infiltration by macrophage cells gene signature in lung adenocarcinoma. Discov Oncol.

[12] van der Kolk B.W., Muniandy M., Kaminska D., Alvarez M., Ko A., Miao Z. (2021). Differential mitochondrial gene expression in adipose tissue following weight loss induced by diet or bariatric surgery. J Clin Endocrinol Metab.

[13] Männistö V., Kaminska D., Käkelä P., Neuvonen M., Niemi M., Alvarez M. (2021). Protein phosphatase 1 regulatory subunit 3B genotype at rs4240624 has a major effect on gallbladder bile composition. Hepatol Commun.

[14] Postic C., Shiota M., Niswender K.D., Jetton T.L., Chen Y., Moates J.M. (1999). Dual roles for glucokinase in glucose homeostasis as determined by liver and pancreatic beta cell-specific gene knock-outs using Cre recombinase. J Biol Chem.

[15] Tsuchida T., Lee Y.A., Fujiwara N., Ybanez M., Allen B., Martins S. (2018). A simple diet- and chemical-induced murine NASH model with rapid progression of steatohepatitis, fibrosis and liver cancer. J Hepatol.

[16] Qin Y., Yu J., Zhang M., Qin F., Lan X. (2019). ZEB1 promotes tumorigenesis and metastasis in hepatocellular carcinoma by regulating the expression of vimentin. Mol Med Rep.

[17] Song Y., Liu C., Liu X., Trottier J., Beaudoin M., Zhang L. (2017). H19 promotes cholestatic liver fibrosis by preventing ZEB1-mediated inhibition of epithelial cell adhesion molecule. Hepatol Baltim Md.

[18] Zhou Y., Zhou B., Pache L., Chang M., Khodabakhshi A.H., Tanaseichuk O. (2019). Metascape provides a biologist-oriented resource for the analysis of systems-level datasets. Nat Commun.

[19] Blue S.M., Yee B.A., Pratt G.A., Mueller J.R., Park S.S., Shishkin A.A. (2022). Transcriptome-wide identification of RNA-binding protein binding sites using seCLIP-seq. Nat Protoc.

[20] Van Nostrand E.L., Pratt G.A., Shishkin A.A., Gelboin-Burkhart C., Fang M.Y., Sundararaman B. (2016). Robust transcriptome-wide discovery of RNA-binding protein binding sites with enhanced CLIP (eCLIP). Nat Methods.

[21] Boyle E.A., Her H.-L., Mueller J.R., Naritomi J.T., Nguyen G.G., Yeo G.W. (2023). Skipper analysis of eCLIP datasets enables sensitive detection of constrained translation factor binding sites. Cell Genom.

[22] Xu S., Nguyen G.G., Naritomi J.T., Kopalle H.M., Yee B.A., Rothamel K.L. (2024). Protocol to process crosslinking and immunoprecipitation data into annotated binding sites. STAR Protoc.

[23] Thorvaldsdóttir H., Robinson J.T., Mesirov J.P. (2013). Integrative Genomics Viewer (IGV): high-performance genomics data visualization and exploration. Briefings Bioinf.

[24] Robinson J.T., Thorvaldsdóttir H., Winckler W., Guttman M., Lander E.S., Getz G. (2011). Integrative genomics viewer. Nat Biotechnol.

[25] Xie Z., Bailey A., Kuleshov M.V., Clarke D.J.B., Evangelista J.E., Jenkins S.L. (2021). Gene set knowledge discovery with Enrichr. Curr Protoc.

[26] Chen E.Y., Tan C.M., Kou Y., Duan Q., Wang Z., Meirelles G.V. (2013). Enrichr: interactive and collaborative HTML5 gene list enrichment analysis tool. BMC Bioinf.

[27] Kuleshov M.V., Jones M.R., Rouillard A.D., Fernandez N.F., Duan Q., Wang Z. (2016). Enrichr: a comprehensive gene set enrichment analysis web server 2016 update. Nucleic Acids Res.

[28] He X., Xie J., Zhang D., Su Q., Sai X., Bai R. (2015). Recombinant adeno-associated virus-mediated inhibition of microRNA-21 protects mice against the lethal schistosome infection by repressing both IL-13 and transforming growth factor beta 1 pathways. Hepatol Baltim Md.

[29] Hu Y., Vinayagam A., Nand A., Comjean A., Chung V., Hao T. (2018). Molecular Interaction Search Tool (MIST): an integrated resource for mining gene and protein interaction data. Nucleic Acids Res.

[30] Choi H., Larsen B., Lin Z.-Y., Breitkreutz A., Mellacheruvu D., Fermin D. (2011). SAINT: probabilistic scoring of affinity purification-mass spectrometry data. Nat Methods.

[31] Snel B., Lehmann G., Bork P., Huynen M.A. (2000). STRING: a web-server to retrieve and display the repeatedly occurring neighbourhood of a gene. Nucleic Acids Res.

[32] Nepusz T., Yu H., Paccanaro A. (2012). Detecting overlapping protein complexes in protein-protein interaction networks. Nat Methods.

[33] Shannon P., Markiel A., Ozier O., Baliga N.S., Wang J.T., Ramage D. (2003). Cytoscape: a software environment for integrated models of biomolecular interaction networks. Genome Res.

[34] Mina A.I., LeClair R.A., LeClair K.B., Cohen D.E., Lantier L., CalR Banks AS. (2018). A web-based analysis tool for indirect calorimetry experiments. Cell Metab.

[35] Huang R., Deng J., Zhu C.-P., Liu S.-Q., Cui Y.-L., Chen F. (2023). Sulodexide attenuates liver fibrosis in mice by restoration of differentiated liver sinusoidal endothelial cell. Biomed Pharmacother Biomedecine Pharmacother.

[36] Bird T.G., Müller M., Boulter L., Vincent D.F., Ridgway R.A., Lopez-Guadamillas E. (2018). TGFβ inhibition restores a regenerative response in acute liver injury by suppressing paracrine senescence. Sci Transl Med.

[37] Matchett K.P., Wilson-Kanamori J.R., Portman J.R., Kapourani C.A., Fercoq F., May S. (2024). Multimodal decoding of human liver regeneration. Nature.

[38] Affò S., Dominguez M., Lozano J.J., Sancho-Bru P., Rodrigo-Torres D., Morales-Ibanez O. (2013). Transcriptome analysis identifies TNF superfamily receptors as potential therapeutic targets in alcoholic hepatitis. Gut.

[39] Asakawa M., Itoh M., Suganami T., Sakai T., Kanai S., Shirakawa I. (2019). Upregulation of cancer-associated gene expression in activated fibroblasts in a mouse model of non-alcoholic steatohepatitis. Sci Rep.

[40] Nakahara H., Ono A., Hayes C.N., Shirane Y., Miura R., Fujii Y. (2025). Multiomics analysis of liver molecular dysregulation leading to nonviral-related hepatocellular carcinoma development. J Proteome Res.

[41] Oki S., Ohta T., Shioi G., Hatanaka H., Ogasawara O., Okuda Y. (2018). ChIP-Atlas: a data-mining suite powered by full integration of public ChIP-seq data. EMBO Rep.

[42] Zou Z., Ohta T., Oki S. (2024). ChIP-Atlas 3.0: a data-mining suite to explore chromosome architecture together with large-scale regulome data. Nucleic Acids Res.

[43] Zou Z., Ohta T., Miura F., Oki S. (2022). ChIP-Atlas 2021 update: a data-mining suite for exploring epigenomic landscapes by fully integrating ChIP-seq, ATAC-seq and Bisulfite-seq data. Nucleic Acids Res.

[44] ENCODE Project Consortium (2012). An integrated encyclopedia of DNA elements in the human genome. Nature.

[45] Guilliams M., Bonnardel J., Haest B., Vanderborght B., Wagner C., Remmerie A. (2022). Spatial proteogenomics reveals distinct and evolutionarily conserved hepatic macrophage niches. Cell.

[46] Rinella M.E., Elias M.S., Smolak R.R., Fu T., Borensztajn J., Green R.M. (2008). Mechanisms of hepatic steatosis in mice fed a lipogenic methionine choline-deficient diet. J Lipid Res.

[47] Gautam J., Aggarwal H., Kumari D., Gupta S.K., Kumar Y., Dikshit M. (2024). A methionine-choline-deficient diet induces nonalcoholic steatohepatitis and alters the lipidome, metabolome, and gut microbiome profile in the C57BL/6J mouse. Biochim Biophys Acta Mol Cell Biol Lipids.

[48] Alshawsh M.A., Alsalahi A., Alshehade S.A., Saghir S.A.M., Ahmeda A.F., Al Zarzour RH. (2022). A comparison of the gene expression profiles of non-alcoholic fatty liver disease between animal models of a high-fat diet and methionine-choline-deficient diet. Molecules.

[49] Rodrigues P.M., Afonso M.B., Simão A.L., Carvalho C.C., Trindade A., Duarte A. (2017). miR-21 ablation and obeticholic acid ameliorate nonalcoholic steatohepatitis in mice. Cell Death Dis.

[50] Campos G., Schmidt-Heck W., De Smedt J., Widera A., Ghallab A., Pütter L. (2020). Inflammation-associated suppression of metabolic gene networks in acute and chronic liver disease. Arch Toxicol.

[51] Liggett J.R., Kang J., Ranjit S., Rodriguez O., Loh K., Patil D. (2022). Oral N-acetylcysteine decreases IFN-γ production and ameliorates ischemia-reperfusion injury in steatotic livers. Front Immunol.

[52] Fazel Modares N., Polz R., Haghighi F., Lamertz L., Behnke K., Zhuang Y. (2019). IL-6 trans-signaling controls liver regeneration after partial hepatectomy. Hepatol Baltim Md.

[53] Luangmonkong T., Suriguga S., Mutsaers H.A.M., Groothuis G.M.M., Olinga P., Boersema M. (2018). Targeting oxidative stress for the treatment of liver fibrosis. Rev Physiol Biochem Pharmacol.

[54] Kim J.-W., Kim Y.J. (2024). The evidence-based multifaceted roles of hepatic stellate cells in liver diseases: a concise review. Life Sci.

[55] Dewidar B., Meyer C., Dooley S., Meindl-Beinker A.N. (2019). TGF-β in hepatic stellate cell activation and liver fibrogenesis-updated 2019. Cells.

[56] Duan N.-N., Liu X.-J., Wu J. (2017). Palmitic acid elicits hepatic stellate cell activation through inflammasomes and hedgehog signaling. Life Sci.

[57] Guo Q., Wu J., Wang Q., Huang Y., Chen L., Gong J. (2023). Single-cell transcriptome analysis uncovers underlying mechanisms of acute liver injury induced by tripterygium glycosides tablet in mice. J Pharm Anal.

[58] Loft A., Alfaro A.J., Schmidt S.F., Pedersen F.B., Terkelsen M.K., Puglia M. (2021). Liver-fibrosis-activated transcriptional networks govern hepatocyte reprogramming and intra-hepatic communication. Cell Metab.

[59] Su Q., Kim S.Y., Adewale F., Zhou Y., Aldler C., Ni M. (2021). Single-cell RNA transcriptome landscape of hepatocytes and non-parenchymal cells in healthy and NAFLD mouse liver. iScience.

[60] Miura A., Hosono T., Seki T. (2021). Macrophage potentiates the recovery of liver zonation and metabolic function after acute liver injury. Sci Rep.

[61] Watanabe T., Niioka M., Ishikawa A., Hozawa S., Arai M., Maruyama K. (2001). Dynamic change of cells expressing MMP-2 mRNA and MT1-MMP mRNA in the recovery from liver fibrosis in the rat. J Hepatol.

[62] Nallagangula K.S., Nagaraj S.K., Venkataswamy L., Chandrappa M. (2018). Liver fibrosis: a compilation on the biomarkers status and their significance during disease progression. Future Sci OA.

[63] Nie Q.-H., Duan G.-R., Luo X.-D., Xie Y.-M., Luo H., Zhou Y.-X. (2004). Expression of TIMP-1 and TIMP-2 in rats with hepatic fibrosis. World J Gastroenterol.

[64] Deng Z., Fan T., Xiao C., Tian H., Zheng Y., Li C. (2024). TGF-β signaling in health, disease, and therapeutics. Signal Transduct Targeted Ther.

[65] Jinnin M., Ihn H., Tamaki K. (2006). Characterization of SIS3, a novel specific inhibitor of Smad3, and its effect on transforming growth factor-beta1-induced extracellular matrix expression. Mol Pharmacol.

[66] Vervoort S.J., Lourenço A.R., Tufegdzic Vidakovic A., Mocholi E., Sandoval J.L., Rueda O.M. (2018). SOX4 can redirect TGF-β-mediated SMAD3-transcriptional output in a context-dependent manner to promote tumorigenesis. Nucleic Acids Res.

[67] Domenicali M., Caraceni P., Giannone F., Baldassarre M., Lucchetti G., Quarta C. (2009). A novel model of CCl4-induced cirrhosis with ascites in the mouse. J Hepatol.

[68] Zhang G., Wang X., Chung T.-Y., Ye W., Hodge L., Zhang L. (2020). Carbon tetrachloride (CCl4) accelerated development of non-alcoholic fatty liver disease (NAFLD)/steatohepatitis (NASH) in MS-NASH mice fed western diet supplemented with fructose (WDF). BMC Gastroenterol.

[69] Katahira J. (2012). mRNA export and the TREX complex. Biochim Biophys Acta.

[70] Strässer K., Masuda S., Mason P., Pfannstiel J., Oppizzi M., Rodriguez-Navarro S. (2002). TREX is a conserved complex coupling transcription with messenger RNA export. Nature.

[71] Weidensdorfer D., Stöhr N., Baude A., Lederer M., Köhn M., Schierhorn A. (2009). Control of c-myc mRNA stability by IGF2BP1-associated cytoplasmic RNPs. RNA N Y N.

[72] Zhan X., Yan C., Zhang X., Lei J., Shi Y. (2018). Structure of a human catalytic step I spliceosome. Science.

[73] Michel K., Roth S., Trautwein C., Gong W., Flemming P., Gressner A.M. (1998). Analysis of the expression pattern of the latent transforming growth factor beta binding protein isoforms in normal and diseased human liver reveals a new splice variant missing the proteinase-sensitive Hinge region. Hepatology.

[74] Presser L.D., Haskett A., Waris G. (2011). Hepatitis C virus-induced furin and thrombospondin-1 activate TGF-β1: role of TGF-β1 in HCV replication. Virology.

[75] Wang H., Li X., Xu X., Li S., Li W., Shi L. (2026). A role of FURIN in promoting TGF-β signaling activation in MAFLD: evidence from UK Biobank proteomic cohort. Int J Biol Macromol.

[76] Eichhorn P.J.A., Rodón L., Gonzàlez-Juncà A., Dirac A., Gili M., Martínez-Sáez E. (2012). USP15 stabilizes TGF-β receptor I and promotes oncogenesis through the activation of TGF-β signaling in glioblastoma. Nat Med.

[77] Li F., Yao J., Yao J., Mou Y., Li D., Wei L. (2025). The role of NEDD4L in metabolic dysfunction-associated fatty liver disease (MAFLD) by regulating ferroptosis via deubiquitinating the TGF-β receptor TβRII. Biochem Cell Biol Biochim Biol Cell.

[78] Li H., Liu G., Wan X., Zhou L., Qin Z.-B., Ma X.-H. (2022). The zinc finger and BTB domain containing protein ZBTB20 regulates plasma triglyceride metabolism by repressing lipoprotein lipase gene transcription in hepatocytes. Hepatol Baltim Md.

[79] Liu G., Zhou L., Zhang H., Chen R., Zhang Y., Li L. (2017). Regulation of hepatic lipogenesis by the zinc finger protein Zbtb20. Nat Commun.

[80] Ni W., Lin S., Bian S., Zheng W., Qu L., Fan Y. (2020). USP7 mediates pathological hepatic de novo lipogenesis through promoting stabilization and transcription of ZNF638. Cell Death Dis.

[81] Lu C., Zhang Y., Miao J., Wei W., Wang Y., Han Y. (2025). Inhibition of ZFP281/ZNF281-RIPK1/RIPK3/MLKL signaling in hepatocytes by pterostilbene relieves hepatic lipometabolic disorder and inflammation in non-alcoholic steatohepatitis. Int Immunopharmacol.

[82] Houten S.M., Wanders R.J.A. (2010). A general introduction to the biochemistry of mitochondrial fatty acid β-oxidation. J Inherit Metab Dis.

[83] Yang L., Roh Y.S., Song J., Zhang B., Liu C., Loomba R. (2014). Transforming growth factor beta signaling in hepatocytes participates in steatohepatitis through regulation of cell death and lipid metabolism in mice. Hepatol Baltim Md.

[84] Liu S.Q., Deng X., Zhu C.P., Cui Y.L., Xie W.F., Zhang X. (2023). Depletion of Tgfbr2 in hepatocytes alleviates liver fibrosis and restores hepatic function in fibrotic mice. J Dig Dis.

[85] Paluschinski M., Kordes C., Vucur M., Buettner V., Roderburg C., Xu H.C. (2023). Differential modulation of miR-122 transcription by TGFβ1/BMP6: implications for nonresolving inflammation and hepatocarcinogenesis. Cells.

[86] Thakral S., Ghoshal K. (2015). miR-122 is a unique molecule with great potential in diagnosis, prognosis of liver disease, and therapy both as miRNA mimic and antimir. Curr Gene Ther.

[87] Culver A., Hamang M., Wang Y., Jiang H., Yanum J., White E. (2023). GDF8 contributes to liver fibrogenesis and concomitant skeletal muscle wasting. Biomedicines.

[88] Holeček M., Vodeničarovová M. (2018). Muscle wasting and branched-chain amino acid, alpha-ketoglutarate, and ATP depletion in a rat model of liver cirrhosis. Int J Exp Pathol.

[89] Charlton M., Krishnan A., Viker K., Sanderson S., Cazanave S., McConico A. (2011). Fast food diet mouse: novel small animal model of NASH with ballooning, progressive fibrosis, and high physiological fidelity to the human condition. Am J Physiol Gastrointest Liver Physiol.

[90] Perez-Riverol Y., Bandla C., Kundu D.J., Kamatchinathan S., Bai J., Hewapathirana S. (2025). The PRIDE database at 20 years: 2025 update. Nucleic Acids Res.

