# Supplementary material for: Zfp697/ZNF697 is an RNA-binding protein that modulates TGF-β1 signaling and hepatic inflammation in liver disease
Source: Mol Metab. 2026 Oct 14;114:102457. doi: 10.1016/j.molmet.2026.102457 (PMC13744273; doi:10.1016/j.molmet.2026.102457)
Supplement: Supplementary file 1 [file mmc6.pdf]

Supplementary Figure 1

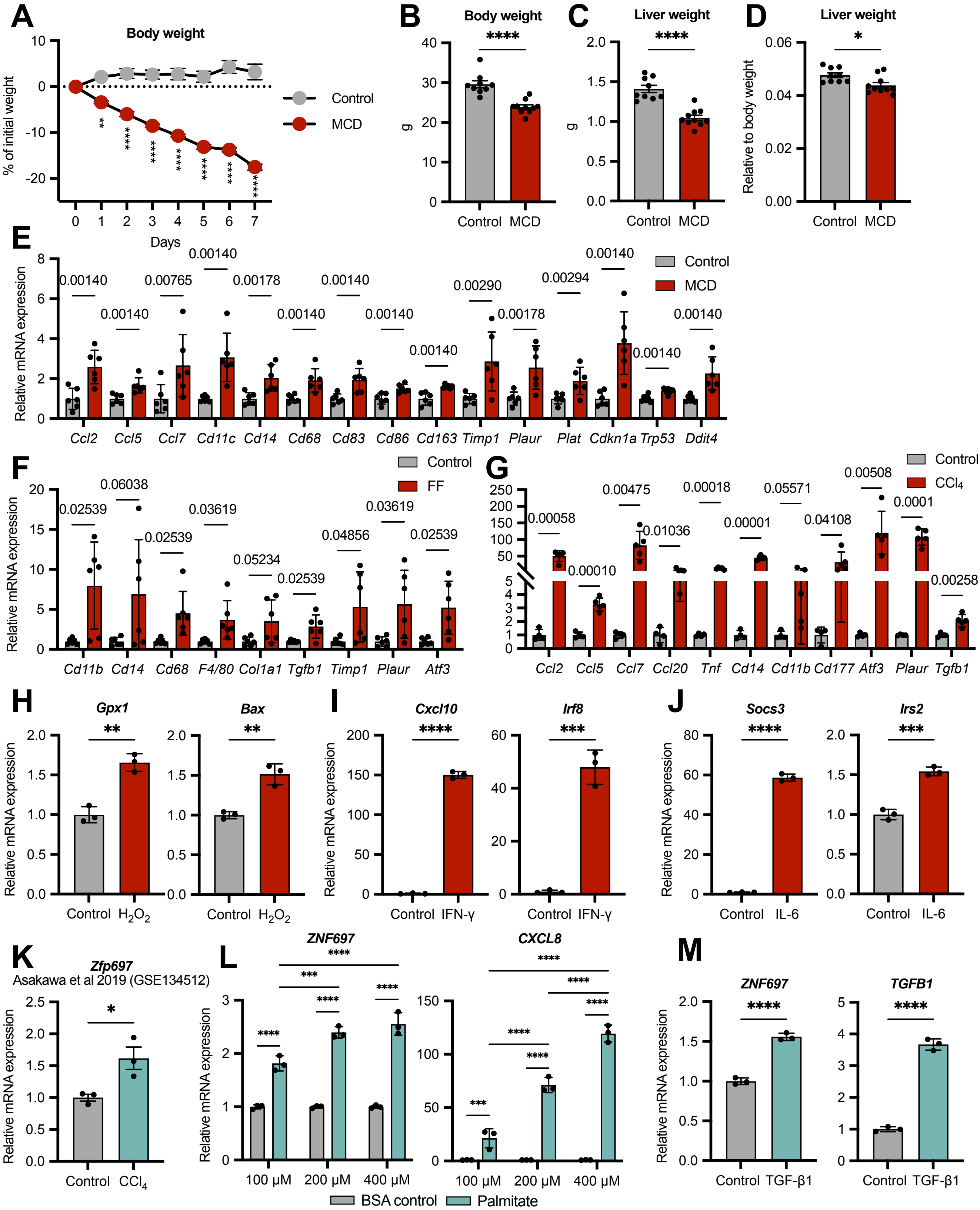

Supplementary Fig. 1: A-E) Related to Fig. 1A: Mice fed a methionine- and choline-deficient (MCD) diet for 7 days (n=9-10). A) Daily body weight monitoring throughout the experiment. B) Final absolute body weight on day 7. Liver weight shown as C) absolute weight and D) relative to body weight. E-G) Related to Fig. 1A-C: qPCR on livers from MCD, fast food (FF), and CCl_4 disease models, respectively (n=4-6). H-J) Related to Fig. 1G-I: mRNA expression data from primary mouse hepatocytes treated for 24 h with H) 0.5 mM H_2O_2 (oxidative stress), I) 10 ng/mL IFN-γ, and J) 40 ng/mL IL-6 (n=3). K) Zfp697 mRNA expression in isolated mouse HSCs from mice treated with CCl_4. Publicity available data (Asakawa et al 2012, GEO: GSE134512) (n=3). L-M) *ZNF697* and control gene expression in LX-2 cells treated for 24 h with L) palmitate and M) 10 ng/mL TGF-β1 (n=3). Statistics: A) Two-way ANOVA with Šidák correction for multiple comparisons. B-K and M) Student's unpaired t-test, for E-G with FDR correction, q values shown, L) Two-way ANOVA with Tukey's test with correction for multiple comparisons. *p<0.05, **p<0.01, ***p<0.001, ****p<0.0001. Error bars represent A-D, K) SEM and E-J, L-M) SD.

Supplementary Figure 2

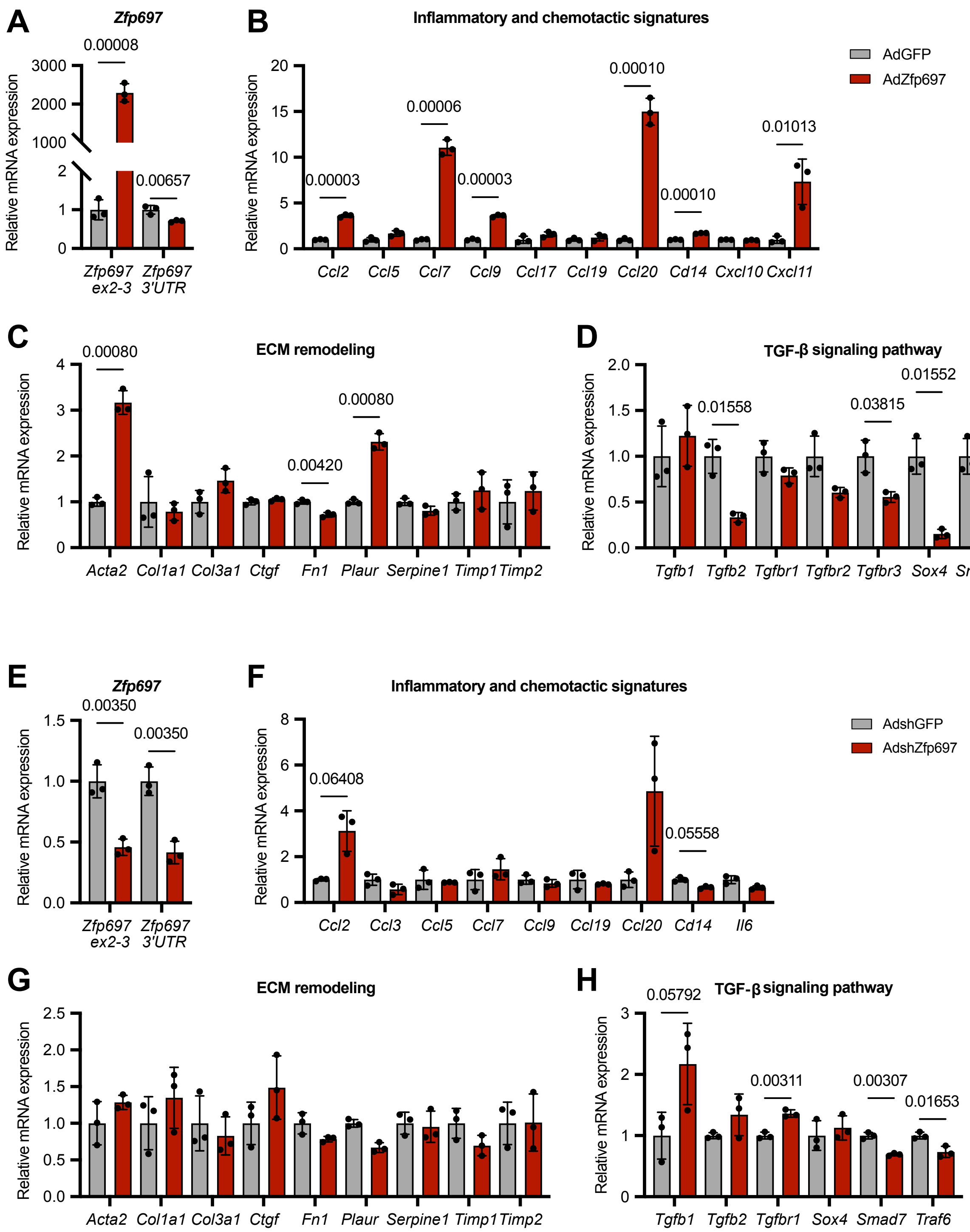

Supplementary Fig. 2: A-D) mRNA expression data from mouse hepatocytes treated with AdZfp697 or AdGFP (n=3). E-H) Primary mouse hepatocytes treated with AdshZfp697 or AdshCtrl for 53 h (n=3). A+E) Zfp697 mRNA expression using primers targeting the exon 2-3 junction or the 3'UTR. B+F) mRNA expression of genes related to inflammation and chemotactic signaling, C+G) ECM remodeling, and D+H) TGF-β signaling. Statistics: Student's unpaired t-test with FDR correction, q-values shown for selected comparisons. Error bars represent SD.

Supplementary Figure 3

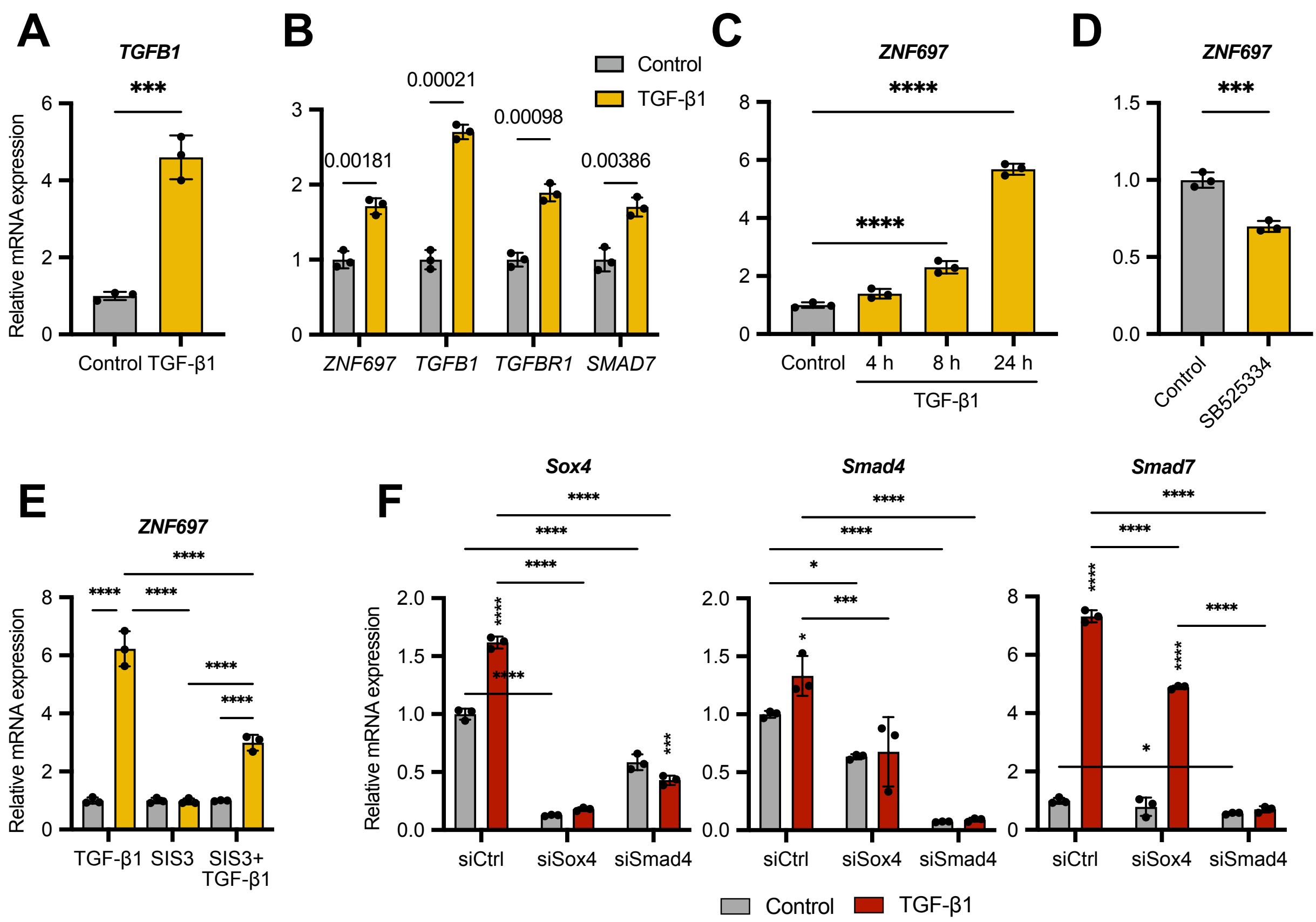

Supplementary Fig. 3: A) TGFB1 mRNA expression in primary human hepatocytes treated with 10 ng/mL TGF-β1 for 24 h (n=3). B) mRNA expression of ZNF697 and classical TGF-β1 target genes in Huh7 cells treated with 10 ng/mL TGF-β1 for 4 h (n=3). C-E) ZNF697 mRNA expression in Huh7 cells treated with C) 10 ng/mL TGF-β1 for 4, 8, or 24 h (n=3), D) 5 μM ALK5 inhibitor SB525334 for 24 h (n=3), E) 10 ng/mL TGF-β1, 10 μM SIS3, or their combination with 30 min SIS3 pre-treatment for 24 h (n=3). F) Sox4, Smad4, and Smad7 mRNA expression in primary mouse hepatocytes treated with siSox4 or siSmad4 for 24 h, followed by 10 ng/mL TGF-β1 stimulation for 24 h (n=3). Statistics: A, D) Student's unpaired t-test, B) Multiple t-tests with FDR correction for multiple testing, q-values indicated, C) One-way ANOVA with Dunnett's correction for multiple comparisons, E, F) Two-way ANOVA with Tukey's test with correction for multiple comparisons. *p<0.05, **p<0.01, ***p<0.001, ****p<0.0001. Error bars represent SD.

Supplementary Figure 4

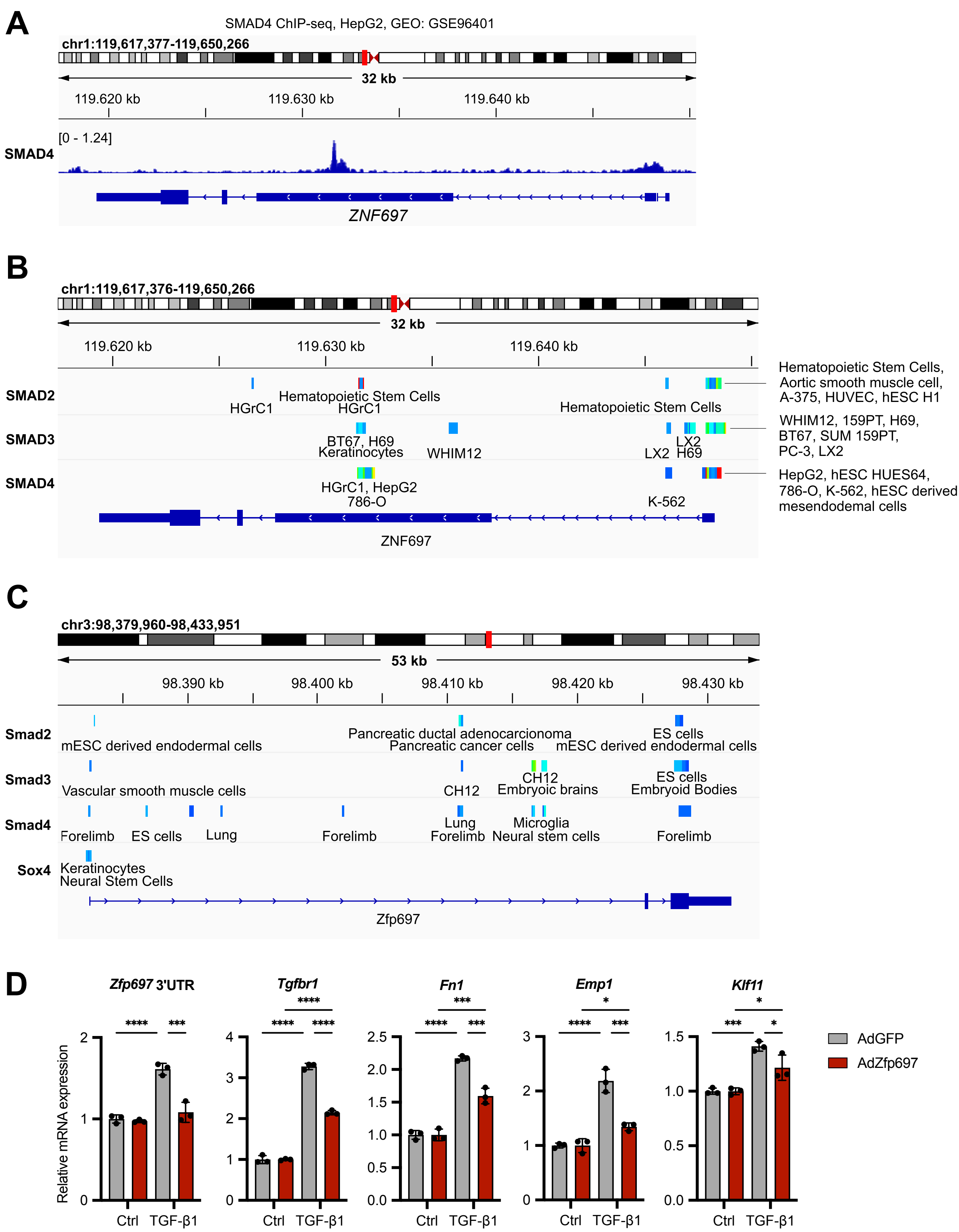

Supplementary Fig. 4: A-C) IGV browser tracks of ChIP-Atlas data showing: A) SMAD4 binding at the *ZNF697* locus in HepG2 cells (ENCODE project, hg38, GEO: GSE96401), B) SMAD2, SMAD3, and SMAD4 binding profiles at the *ZNF697* locus in various human cell lines (hg38), and C) Smad2, Smad3, Smad4, and Sox4 binding profiles at the *Zfp697* locus in various mouse tissues and cell lines (mm10). D) Related to Fig. 3F-G: mRNA expression of *Zfp697* (3'UTR) and other TGF-β1 responsive genes in primary mouse hepatocytes pre-treated for 24 h with AdZfp697 or AdGFP, followed by 10 ng/mL TGFβ-1 for 24 h (n=3). Same data as in Fig. 3G, shown here with expressions relative to control treatment. Statistics: D) Two-way ANOVA with Tukey's test with correction for multiple comparisons. *p<0.05, **p<0.01, ***p<0.001, ****p<0.0001. Error bars represent SD.

Supplementary Figure 5

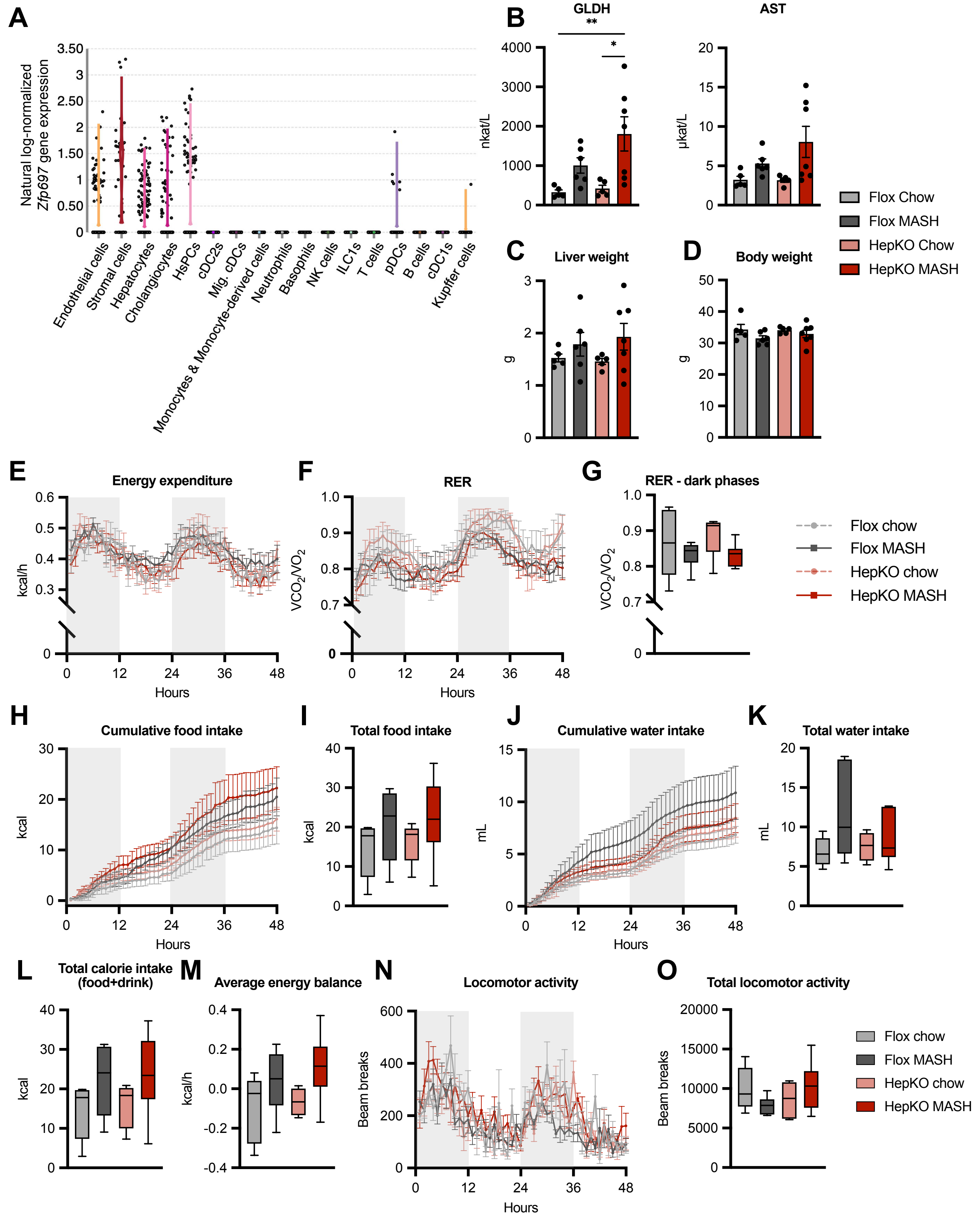

Supplementary Fig. 5: A) *Zfp697* expression across mouse liver cell types based on publicly available single-cell RNA-seq data. Data are from Liver Cell Atlas (livercellatlas.org) Guilliams et al 2022. B) Plasma levels of GLDH and AST (n=5-7). C-D) Absolute liver weight and body weight (n=5-7). E-O) Indirect calorimetry over 48 h (two light/dark cycles). Shaded areas mark dark photoperiods (18:00-6:00), solid lines indicate group hourly averages (n=5-7). E) Hourly average of energy expenditure. F-G) Respiratory exchange ratio shown as 48 h curve and dark-phase average. H) Cumulative food intake and I) total food intake (n=5-6). J) Cumulative water intake and K) total water intake (n=4-7). L) Total calorie intake from food and drink (n=4-6). M) Average energy balance over 48 h (n=4-6). N) Hourly average locomotor activity and O) total activity (n=5-7). Statistics: B-D) One-way ANOVA. E-O) ANCOVA or one-way ANOVA: no significant differences observed. *p<0.05, **p<0.01. Error bars represent SEM.

Supplementary Figure 6

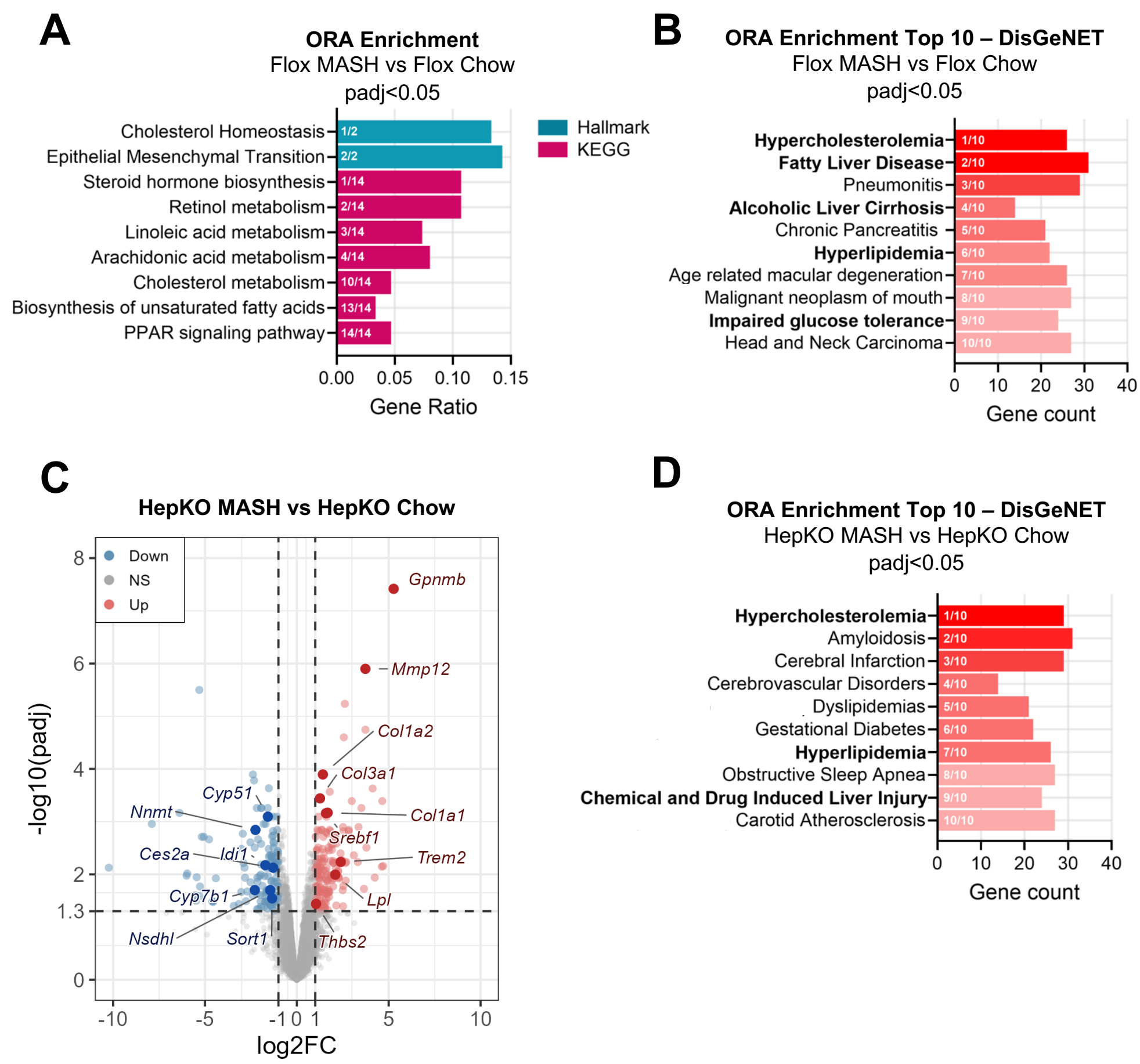

Supplementary Fig. 6: RNA-seq analysis of liver tissue from chow- and MASH-fed Flox and HepKO mice. A) Over-representation analysis (ORA) of significantly differentially expressed genes identified in Flox MASH versus Flox Chow. Enriched Hallmark and KEGG pathways are shown as gene ratio. B) DisGeNET disease enrichment analysis of significantly differentially expressed genes identified in Flox MASH versus Flox Chow. C) Volcano plot showing differentially expressed genes in HepKO MASH versus HepKO Chow. Selected significantly upregulated and downregulated genes are indicated. D) DisGeNET disease enrichment analysis of significantly differentially expressed genes identified in HepKO MASH versus HepKO Chow. Only significantly enriched disease annotations are shown.

Supplementary Figure 7

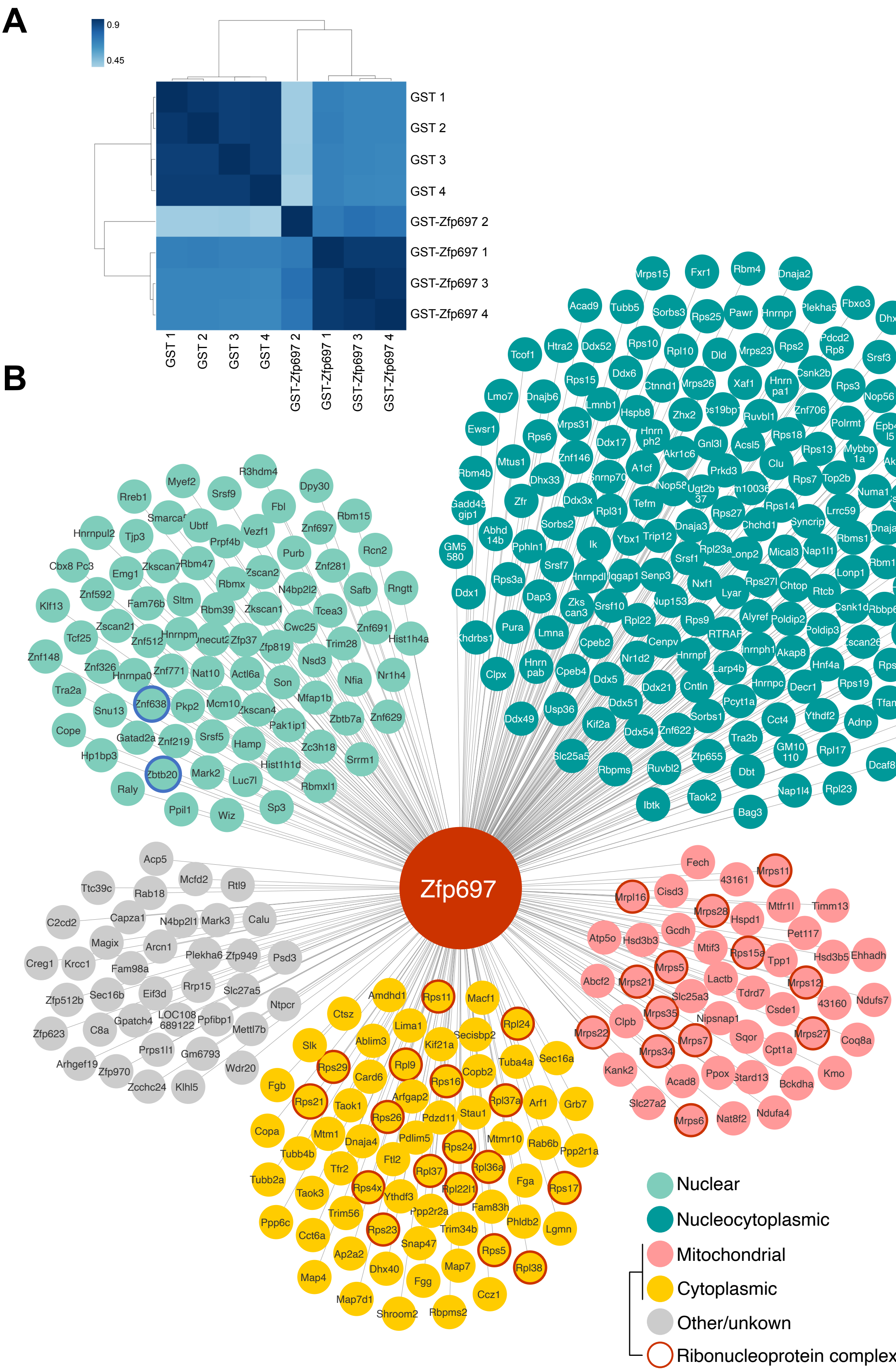

Supplementary Fig. 7: A) Related to Fig. 5B: Clustermap of pairwise Pearson correlation coefficients in all 4 GST control and 4 GST-Zfp697 samples, including the GST-Zfp697 sample 2, which was excluded from downstream analyses. B) Zfp697 protein interaction partners grouped into five categories based on their curated subcellular localization: nuclear, nucleocytoplasmic, mitochondrial, cytoplasmic, or unknown. Open blue circles highlight examples referenced in the main text.

Supplementary Figure 8

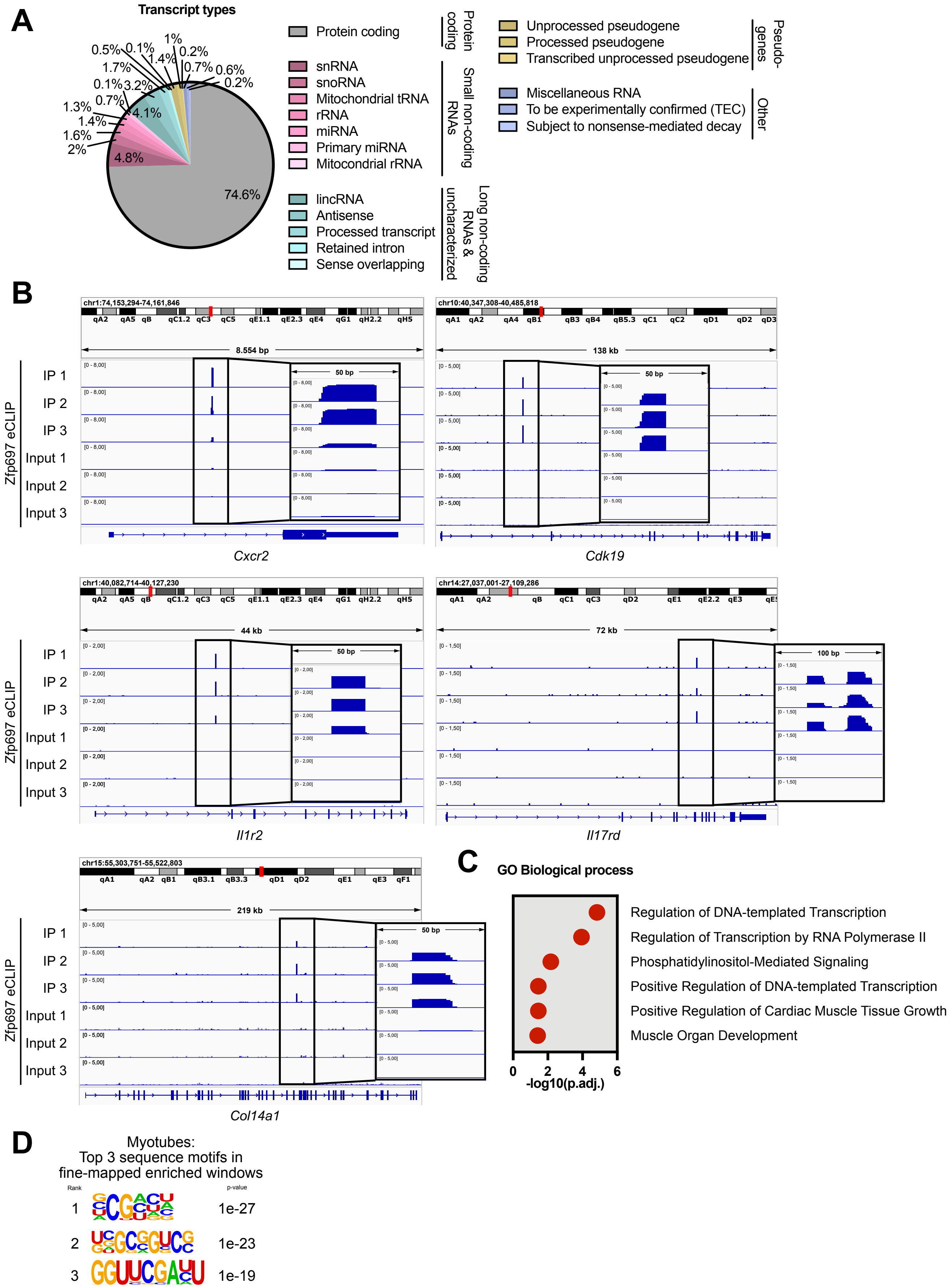

Supplementary Fig. 8: A) Classification of RNA transcripts types interacting with Zfp697. B) IGV browser tracks of eCLIP data showing representative examples of Zfp697-RNA transcripts interactions. C) Enrichr GO Biological Process enrichment analysis of Zfp697-associated RNAs. D) Top three sequence motifs identified in enriched windows in primary mouse myotubes, based on HOMER *de novo* motif analysis on fine-mapped enriched windows.

Click here to access/download
Supplementary material
Table S1 KOBS.xlsx

Click here to access/download
Supplementary material
Table S2 RNA-seq.xlsx

Click here to access/download
Supplementary material
Table S3 Protein.xlsx

Click here to access/download
Supplementary material
Table S4 eCLIP.xlsx

Click here to access/download
Supplementary material
Table S5 Primers.docx
