# Supplementary material for: Zfp697/ZNF697 is an RNA-binding protein that modulates TGF-β1 signaling and hepatic inflammation in liver disease
Source: Mol Metab. 2026 Oct 14;114:102457. doi: 10.1016/j.molmet.2026.102457 (PMC13744273; doi:10.1016/j.molmet.2026.102457)
Supplement: Supplementary file 2 [file mmc5.docx]

**Table S4: Primers**

| **Target** | **Species** | **Forward (5’ - 3’)** | **Reverse (5’ - 3’)** |
| --- | --- | --- | --- |
| *Acox1* | Mouse | TGAGCTTCATGCCCTCACAG | TGCTGTGAGAATAGCCGTGC |
| *Acta2* | Mouse | CCAGGCATTGCTGACAGGAT | AAGGTAGACAGCGAAGCCAG |
| ACTA2 | Human | AGCGTGGCTATTCCTTCGTT | GTGGTTTCATGGATGCCAGC |
| *Atf3* | Mouse | CGTCAACAACAGACCCCTGG | TGTTTCGACACTTGGCAGCA |
| *Bax* | Mouse | TGCTAGCAAACTGGTGCTCA | TCTTGGATCCAGACAAGCAGC |
| *Btg2* | Mouse | GTGGGTTTCCTCTCCAGTCTC | ACGGATACAGCGATAGCCAG |
| *Ccl17* | Mouse | GCTCGAGCCACCAATGTAGG | GCATCCCTGGAACACTCCAC |
| *Ccl19* | Mouse | TCGGCCTCTCAGATTCTTGC | GCTGGGAAGGTCCAGAGAAC |
| *Ccl2* | Mouse | TTAAAAACCTGGATCGGAACCAA | GCATTAGCTTCAGATTTACGGGT |
| *Ccl20* | Mouse | ACTGTTGCCTCTCGTACATACA | GAGGAGGTTCACAGCCCTTTT |
| *Ccl3* | Mouse | TTCTCTGTACCATGACACTCTGC | CGTGGAATCTTCCGGCTGTAG |
| *Ccl5* | Mouse | GCTGCTTTGCCTACCTCTCC | TCGAGTGACAAACACGACTGC |
| *Ccl7* | Mouse | CACCAGTAGTCGGTGTCCCT | GCTATAGCCTCCTCGACCCA |
| *Ccl9* | Mouse | AGCCTTTTCATACTGCCCTCT | TTCAGACTGCTCTGGACTTCTT |
| *Cd11b* | Mouse | NA | NA |
| *Cd11c* | Mouse | CTGGATAGCCTTTCTTCTGCTG | GCACACTGTGTCCGAACTCA |
| *Cd14* | Mouse | CTCTGTCCTTAAAGCGGCTTAC | GTTGCGGAGGTTCAAGATGTT |
| *Cd163* | Mouse | CTGGCGGGTGGTGAAAACA | CCAGCCGTTACTGCACACTG |
| *Cd177* | Mouse | GCTGCCAAGACACACTGATG | CAGTTCTGTGCTCCGTGACT |
| *Cd40l* | Mouse | AACGTGACTGAAGCAAGCCAAGTG | AGTTCAACACAGGGCAGGTCCTAA |
| *Cd68* | Mouse | TGTCTGATCTTGCTAGGACCG | GAGAGTAACGGCCTTTTTGTGA |
| *Cd83* | Mouse | GCAGCTCTCCTATGCAGTG | GTGTTTTGGATCGTCAGGGAATA |
| *Cdkn1a* | Mouse | CTAGGGGAATTGGAGTCAGGC | AACAGGTCGGACATCACCAG |
| CDKN1A | Human | TGGAGACTCTCAGGGTCGAAA | GGCGTTTGGAGTGGTAGAAATC |
| *Col1a1* | Mouse | CTGACGCATGGCCAAGAAG | GTGCCATTGTGGCAGATACAG |
| *Col3a1* | Mouse | GGCACAGCAGTCCAACGTA | TTGACATGGTTCTGGCTTCCA |
| COL3A1 | Human | GGAGCTGGCTACTTCTCGC | GGGAACATCCTCCTTCAACAG |
| *Ctgf* | Mouse | AGACCTGTGCCTGCCATTAC | ACGCCATGTCTCCGTACATC |
| *Cxcl10* | Mouse | ATGACGGGCCAGTGAGAATG | ATGATCTCAACACGTGGGCA |
| *Cxcl11* | Mouse | ACAGGAAGGTCACAGCCATAG | ATCTCTGCCATTTTGACGGCT |
| CXCL8 | Human | TGTGAAGGTGCAGTTTTGCCAAGG | GTTGGCGCAGTGTGGTCCACTC |
| *Cyp8b1* | Mouse | CTAGGGCCTAAAGGTTCGAGT | GTAGCCGAATAAGCTCAGGAAG |
| CYP8B1 | Human | CTGGAGACCAAGCAGTCCTTTG | GATACTCCTGCCCACTGGACAT |
| *Ddit4* | Mouse | CTTGTCCGCAATCTTCGCTG | TGGCTGCTAAGGTCCGAATG |
| *Emp1* | Mouse | TCCCTGTCCTACGGCAATGAAG | CTGGAACACGAAGACCACAAGG |
| *F4/80* | Mouse | TCACTGTCTGCTCAACCGTC | AGAAGTCTGGGAATGGGAGC |
| *Fn1* | Mouse | AATGGAAAAGGGGAATGGAC | CTCGGTTGTCCTTCTTGCTC |
| *Gadd45a* | Mouse | CCGGGAAAGTCGCTACATGG | TCCATTCGGATGCCATCACC |
| *Gpx1* | Mouse | GGGACTACACCGAGATGAACGA | ACCATTCACTTCGCACTTCTCA |
| *Hprt* | Mouse | AGTCCCAGCGTCGTGATTAG | TTTCCAAATCCTCGGCATAATGA |
| HPRT | Human | GAAAAGGACCCCACGAAGTGT | AGTCAAGGGCATATCCTACAACA |
| *Il6* | Mouse | TAGTCCTTCCTACCCCAATTTCC | TTGGTCCTTAGCCACTCCTTC |
| *Irf8* | Mouse | GATCGAACAGATCGACAGCA | GCTGGTTCAGCTTTGTCTCC |
| *Irs2* | Mouse | AACCTGAAACCTAAGGGACTGG | CGGCGAATGTTCATAAGCTGC |
| *Klf11* | Mouse | CATGGACATTTGTGAGTCGATCC | CCTTTGGTAGATCAGGTGCAG |
| *Mmp2* | Mouse | ACGATGATGACCGGAAGTGG | GTGTAGATCGGGGCCATCAG |
| *Mmp9* | Mouse | GGATCCCCAGAGCGTCATTC | TTGGAAACTCACACGCCAGA |
| *Myh9* | Mouse | GTGAGAAGGAGACAAAGGCG | GACTTCTCCAGCTCGTGGAC |
| *Nedd9* | Mouse | CCACCCTCCTACCAGAATCA | ATACCCCTTGAGTGCTGTGG |
| *Pdk4* | Mouse | AGGGAGGTCGAGCTGTTCTC | GGAGTGTTCACTAAGCGGTCA |
| *Plat* | Mouse | TCGGGACACAGAAGAAACGG | TTGTCTGCGTTGGCTCATCT |
| *Plaur* | Mouse | GCAACCAGACCTTTCACTTCC | AACACTGGAAGCCATTCGGT |
| *Plk3* | Mouse | ACCAAGAGCCTGTTTGGCAG | ATCTGGATGACCGATGGATGTG |
| *Serpine1* | Mouse | CAATGACTGGGTGGAAAGGCA | GGCGTGTCAGCTCGTCTAC |
| *Slc16a2* | Mouse | GTGCTCTTGGTGTGCATTGG | CCGAAGTCCCGGCATAGG |
| *Slc7a2* | Mouse | TCTATGTTCCCCTTACCCCGA | TGACTGCCTCTTACTCACTCTT |
| *Smad4* | Mouse | AAGGTGACGTTTGGGTCAGG | ACCTTTATATACGCGCTTGGGT |
| *Smad7* / SMAD7 | Mouse / Human | ACGAATTATCTGGCCCCTGG | AGCCATTCCCCTGAGGTAGA |
| *Socs3* | Mouse | ATGGTCACCCACAGCAAGTTT | TCCAGTAGAATCCGCTCTCCT |
| *Sox4* | Mouse | CCTCGCTCTCCTCGTCCT | TCGTCTTCGAACTCGTCGT |
| *Tbp* | Mouse | CCTTGTACCCTTCACCAATGAC | ACAGCCAAGATTCACGGTAGA |
| TGFB1 | Human | CCCAGCATCTGCAAAGCTC | GTCAATGTACAGCTGCCGCA |
| *Tgfb1* | Mouse | ATGCTAAAGAGGTCACCCGC | TGCTTCCCGAATGTCTGACG |
| *Tgfb2* | Mouse | GAAGACCCCACATCTCCTGC | TGCCCCAGCACAGAAGTTAG |
| *Tgfbr1* | Mouse | CCTCGAGACAGGCCATTTGTA | AGGTCCTGTAGTTGGGAGTTCT |
| TGFBR1 | Human | ATCACCTGGCCTTGGTCCTGTGG | GGTCCTCTTCATTTGGCACTCGATG |
| *Tgfbr2* | Mouse | NA | NA |
| *Tgfbr3* | Mouse | NA | NA |
| *Timp1* | Mouse | GTAAGGCCTGTAGCTGTGCC | AGGTGGTCTCGTTGATTTCTGG |
| *Timp2* | Mouse | CCTGGGACACGCTTAGCATC | GGATCATGGGACAGCGAGTG |
| *Tnfa* | Mouse | CAGGCGGTGCCTATGTCTC | CGATCACCCCGAAGTTCAGTAG |
| *Traf6* | Mouse | TTGGTTGTGAAGTCTCAGCGT | ACACCTTCTTCTGGCTTTCGT |
| *Trp53* | Mouse | TGCTCACCCTGGCTAAAGTTC | ATCCGACTGTGACTCCTCCA |
| *Trp53inp1* | Mouse | CTATGCTGTGCACAACTCCTG | GTGCTTCCCCATTTCACTCTG |
| *Zfp697 3’UTR* | Mouse | TGGCGTGAAGTAATGGCAGT | TGCTAAGGGGTTTGGCTCAG |
| *Zfp697ex2-3* | Mouse | GGACTCCAACCCACAGGATG | TTTCTTCCTCACTCAGCCGC |
| ZNF697 | Human | TTGCTATGCCTGGTCTCGTC | AGCTTCACTCTCCACTCCCT |
